# Feruloylmonotropeins: promising natural antioxidants in *Paederia scandens*†

**DOI:** 10.1039/d3ra00458a

**Published:** 2023-02-20

**Authors:** Nguyen Quang Trung, Nguyen Thi Thu Thanh, Nguyen Thi Hoa, Adam Mechler, Quan V. Vo

**Affiliations:** a The University of Danang – University of Science and Education Da Nang 550000 Vietnam nqtrung.quatest2@gmail.com; b Quality Assurance and Testing Center 2 Da Nang 550000 Vietnam; c Le Thanh Phuong High School An My Phu Yen 621640 Vietnam; d The University of Danang – University of Technology and Education Danang 550000 Vietnam vvquan@ute.udn.vn; e Department of Biochemistry and Chemistry, La Trobe University Victoria 3086 Australia

## Abstract

*Paederia scandens (Lour.)* is a widely used medicinal herb in Vietnam, China, India, and Japan for the treatment of a variety of conditions, including toothache, chest pains, piles, and spleen inflammation. There is broad interest in identifying the composition of its extracts and confirming their numerous biological activities, including anti-nociceptive, antiviral, and anticancer properties. Two iridoid glucosides obtained from the MeOH extract of *P. scandens*, 6′-O-*E*-feruloylmonotropein (6-FMT) and 10′-O-*E*-feruloylmonotropein (10-FMT), are potential antioxidants based on their structure. In this study, the hydroperoxyl scavenging activity of 6-FMT and 10-FMT was examined *in silico* by using density functional theory. These FMTs are predicted to be weak antioxidants in non-polar environments, whereas a good HOO˙ scavenging activity is expected in polar environments (pH = 7.4) with *k*_overall_ = 3.66 × 10^7^ M^−1^ s^−1^ and 9.45 × 10^6^ M^−1^ s^−1^, respectively. This activity is better than many common antioxidants such as trolox and nearly equivalent to ascorbic acid and resveratrol. The hydroperoxyl scavenging activity was exerted mainly by the di-anion form of FMTs in water at physiological pH following the single electron transfer mechanism. The results suggest that FMTs are promising natural antioxidants in aqueous physiological environments.

## Abstract 2

Feruloylmonotropeins (FMTs), derived from *Paederia scandens*, are expected to be potent radical scavengers in physiological aqueous environments.

## Graphical abstract

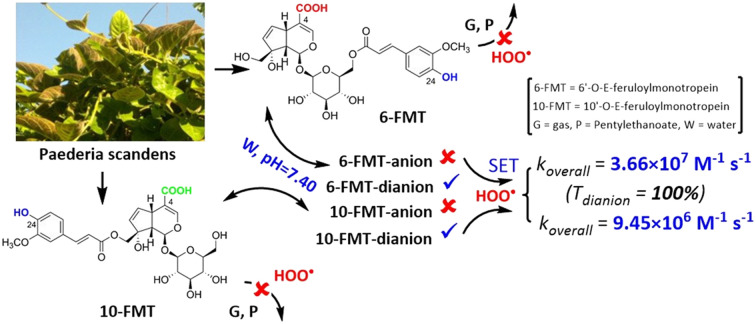

## 1. Introduction

*Paederia scandens* (*Lour.*) (*P. scandens*) is a species of the Rubiaecae family, which is widely distributed in the southern region of the Korean peninsula, Vietnam, India, China, Japan, the Philippines, and the USA^1^*P. scandens* is a common dietary herb, but it is also a medicinal plant. The roots and aerial parts of this plant are used in traditional medicine to treat toothache, chest pains, piles, inflammation of the spleen, as well as for simple effects as emetic and diuretic.^2^ Recent works on the constituents and bioactivities of extracts from parts of *P. scandens* showed that extracts have anti-nociceptive, antiviral, antitumor, and anti-inflammatory activities.^3–5^ In the extracts several bioactive substances were identified such as iridoid glucosides,^1,6,7^ volatile oils,^8–10^ flavonoids,^11,12^ glucosides,^13–15^ and quinones.^13,16,17^

**Fig. 1:**
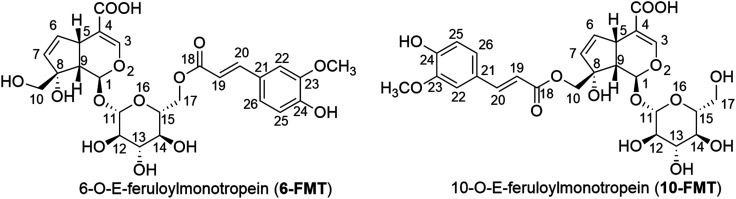
Molecular structure and atomic numbering of FMT.

Two iridoid glucosides: 6′-O-*E*-feruloylmonotropein (6-FMT) and 10′-O-*E*-feruloylmonotropein (10-FMT) (Fig. 1) were isolated from the MeOH extract of *P. scandens*.^6^ Recent works also elaborated on antioxidant activity of the *P. scandens* extracts, showing among others the elimination of hydroxyl free radicals, and antioxidant enzyme activity.^18,19^ However, there is no tangible link thus far between the presence of iridoid glucosides in the extracts and their antioxidant activity, which is highly likely based on structural characteristics.

In the past few years, numerous studies were conducted on bioactivities of natural compounds using a computational approach.^20–25^ From the kinetic and thermodynamic parameters, the main mechanistic pathways of radical scavenging, as well as the capacity of radical scavenging activity in different media (gas phase, polar and non-polar solvent at physiological pH) were described.^26,27^ Those calculated results were useful for confirming the results of the earlier experimental studies on antioxidant activities of natural compounds. In this paper, we use this approach to investigate the antiradical properties of 6-FMT and 10-FMT (Fig. 1) through three main mechanisms: formal hydrogen transfer (FHT), single electron transfer followed by proton transfer (SETPT), and sequential proton loss electron transfer (SPLET).

## 2. Computational details

Thermochemical parameters: bond dissociation energies (BDEs), ionization energies (IEs) and proton affinities (PAs), together with kinetic parameters including activation energies Δ*G*^‡^ (kcal mol^−1^), tunneling corrections (*κ*) and rate constants (*k*) in the gas phase and in physiological environments (water for the aqueous solution and pentyl ethanoate for lipid medium) were calculated by M06-2X functional.^28–32^ The quantum mechanics based test for overall free radical scavenging activity (QM-ORSA) protocol^23,28,33–36^ was used for the kinetic calculations.

The rate constant (*k*) was computed following the transition state theory (TST) and 1 M standard state at 298.15 K by the formula (1):^27,29,36–41^1
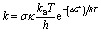
where s is the reaction symmetry number, ^42,43^*κ* contains tunneling corrections which were computed using Eckart barrier,^44^*k*_B_ is the Boltzmann constant, *h* is the Planck constant, Δ*G*^‡^ is Gibbs free energy of activation. The details of the method are shown in Table S3, ESI.†^27^ All DFT calculations in this work were computed using Gaussian 16 package.^45^ The good performance of DFT with the M06-2X functional for thermodynamics and kinetics calculations has been proven by prior studies.^31,32^

Due to the large molecules (∼70 atoms), the thermodynamic data were calculated at M06-2X/6-311++G(d,p)//M06-2X/6-31+G(d)) level of theory, whereas the kinetic results were investigated by using the M06-2X/6-31+G(d). This level was sufficient for geometry optimization with acceptable accuracy, confirmed by previous studies.^22,34,46^

## 3. Results and discussion

### 3.1. The HOO˙ radical scavenging of feruloylmonotropeins in the gas phase

#### 3.1.1. Thermodynamic evaluation

The three main mechanisms of antioxidant activity were defined in previous studies; these are the FHT, SPLET, and SETPT reactions.^24,47^ It is not trivial to identify which mechanism is more favorable for a specific compound, therefore as initial screening, the thermodynamic parameters are calculated that define the first stage, essentially approximating the energy barrier of the initial step. Thus, bond dissociation enthalpy (BDE), proton affinity (PA), and ionization energies (IE) were computed in the gas phase.^22^ To save computing resource, the BDE and PA of each bonds were pre-computed at the M06-2X/6-31 G(d) level of theory, then the bonds with the lowest values were re-computed with a larger basis set. The results are shown in Table 1.

**Table 1:** The calculated BDE, IE, and PA (in kcal mol^−1^) of FMT and Δ*G*^o^ of the first step of the FMT + HOO˙ reactions

Positions	BDE	Δ*G*^o^	PA	Δ*G*^o^	IE	Δ*G*^o^
6FMT–O4–H	116.0	30.0	321.0	202.9	187.7	163.0
6FMT–C5–H	82.6	–2.9				
6FMT–O24–H	97.6	11.0	333.6	215.6		
10FMT–O4–H	113.8	24.9	329.4	211.3	174.3	150.8
10FMT–C5–H	78.8	–9.4				
10FMT–O24–H	87.0	–1.3	325.9	207.0		

The above results suggest that the lowest BDE value of O–H bonds was at C5 and O24 for both studied compounds. The BDE values of these positions of 6-FMT are 82.6 kcal mol^−1^ and 97.6 kcal mol^−1^, and that of 10-FMT are 78.8 kcal mol^−1^ and 87.0 kcal mol^−1^, for the bonds at C5–H and O24–H, respectively. These O24-BDE values are higher than that of typical antioxidants such as viniferifuran (82.7 kcal mol^−1^),^48^ resveratrol (83.9 kcal mol^−1^),^48^ and vanillic acid (85.2 kcal mol^−1^);^49^ thus the radical scavenging activity of these positions is expected to be weaker than the reference antioxidants.

The Gibbs free energy changes of the first step of the FMT + HOO˙ reaction following each pathway *i.e.* FHT, SET (single electron transfer-SET, the first step for SETPT mechanism), and proton loss (PL, the first step of SPLET mechanism) were also calculated to confirm the favored pathway of FMT antiradical activity. The results show that the reaction at 6-FMT–C5–H, 10-FMT–C5–H, and 10-FMT–O24–H are spontaneous due to the Δ*G*^o^ < 0, except for 6-FMT–O24–H position with Δ*G*^o^ = 11.0. Whereas the Δ*G*^o^ of the reaction following the SP and SET mechanism are much higher than that of FHT mechanism. The above data show that the radical scavenging activity of FMT in the gas phase will not follow SETPT nor SPLET mechanism. Thus, the kinetic study of FHT pathway of C5–H and O24–H positions should be investigated in the next section.

##### 3.1.1.1 Kinetic study

**Fig. 2:**
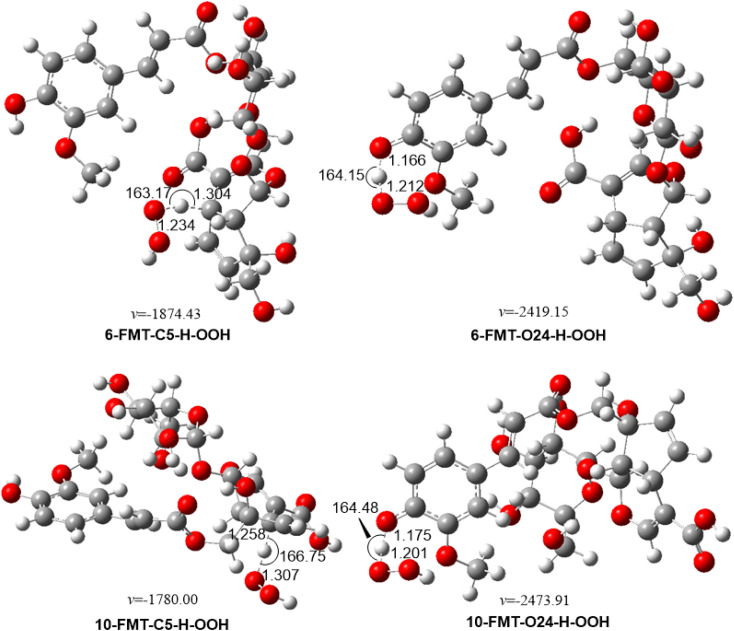
Optimized geometries of TSs between the studied compounds and HOO˙ radical in the gas phase following the FHT pathway.

From the data above, the preferred mechanism is FHT at C5–H and O24–H position. Accordingly, the kinetic parameters were calculated for the lowest BDE values of each type of bond. The results are presented in Table 2 and Fig. 2.

**Table 2:** Calculated activation Gibbs free energies (Δ*G*^‡^, kcal mol^−1^), tunneling corrections (*k*), *k*_Eck_, *k*_overall_ (M^−1^ s^−1^) and branching ratios (*Γ*, %) for the HOO˙ scavenging of the FMT in the gas phase

Comp.	Mechanisms	Positions	Δ*G*^‡^	*κ*	*k*_Eck_	*Γ*
6-FMT	FHT	C5–H	21.0	457.6	1.20	0.0
		O24–H	17.2	3322.0	5.30 × 10^3^	100.0
	*k*_overall_				5.30 × 10^3^	
10-FMT	FHT	C5–H	16.2	24.1	1.99 × 10^2^	76.7
		O24–H	17.6	72.7	6.02 × 10^1^	23.3
	*k*_overall_				2.59 × 10^2^	

According to the above data, both 6-FMT and 10-FMT are weak antioxidants, with *k*_overall_ = 5.30 × 10^3^ and 2.59 × 10^2^ M^−1^ s^−1^, respectively. The O24–H contribute 100% of the antioxidant activity of 6-FMT, while that of 10-FMT is only 23.3%. The results show that the substitution of ferulic acid at the 17 position of pyranosyl moiety (6-FMT) causes the higher HOO˙ radical scavenging activity of FMT than at the 10 position of iridoid skeleton moiety (10-FMT). Besides, those low reaction rates correspond to the high BDE values in the prior section.

### 3.2. The HOO˙ radical scavenging of feruloylmonotropeins in the physiological environments

#### 3.2.1. Thermodynamic evaluation

Prior studies established that the antioxidant activity of phenolic compounds in solvent environments is greatly enhanced by the deprotonation of OH bonds.^29,31,50^ Thus, the contribution of each protonation state, including neutral molecular (HA), anion (HA^−^), and dianion (A^2−^) states should be calculated. Those studies also showed that the antioxidant activity of the phenolic compounds mainly follows the SPLET mechanism, where the spontaneous dissociation of acidic moieties eliminates the activation energy barrier of the first step of the reaction (PL) and so the activity proceeds to the second step (SET), rendering the reaction energetically preferred. Accordingly, the deprotonation of 6-FMT and 10-FMT in water should be investigated. The calculated thermodynamic parameter (BDE, PA, IE) of FMT and Δ*G*^o^ of the first step of the FMT + HOO˙ reactions in pentyl ethanoate and water are presented in Table 3 and 4.

**Table 3:** The calculated thermodynamic parameter (BDE, IE, PA, in kcal mol^−1^) of FMT and Δ*G*^o^ of the first step of the FMT + HOO˙ reactions in pentyl ethanoate

Positions	BDE	Δ*G*^o^	PA	Δ*G*^o^	IE	Δ*G*^o^
6-FMT–O4–H			298.6	98.2	153.3	46.1
6-FMT–C5–H	79.2	–16.8				
6-FMT–O24–H	93.9	–3.1	308.8	108.4		
10-FMT–O4–H			298.1	97.6	141.7	35.7
10-FMT–C5–H	75.7	–24.0				
10-FMT–O24–H	84.7	–15.1	299.5	98.2		

**Table 4:** The computed thermodynamic parameter (BDE, IE, PA, in kcal mol^−1^) of FMT and Δ*G*^o^ of the first step of the FMT + HOO˙ reactions in water

Positions	BDE	Δ*G*^o^	PA	Δ*G*^o^	IE	Δ*G*^o^
6-FMT–O4–H			40.5	35.9	116.5	34.4
6-FMT–C5–H	76.6	–20.5				
6-FMT–O24–H	90.9	–7.2	50.0	45.5		
10-FMT–O4–H			35.7	31.2	107.1	26.3
10-FMT–C5–H	75.5	–21.9				
10-FMT–O24–H	81.7	–15.9	38.7	33.4		

As per Table 3, the BDE values of the C5–H and O24–H position of both studied substances in pentyl ethanoate are lower than in the gas phase, corresponding to negative free Gibbs energies. The lowest PA values of 6-FMT and 10-FMT range from 298.6 kcal mol^−1^ to 308.8 kcal mol^−1^, and from 298.1 kcal mol^−1^ to 299.5 kcal mol^−1^, respectively. The IE value of 6-FMT is 153.3 kcal mol^−1^ and that of 10-FMT is 141.7 kcal mol^−1^. The computed data suggested that the HOO˙ radical scavenging of 6-FMT and 10-FMT in apolar environments do not follow either the SPLET or SETPT mechanism, and those pathways can be omitted in the kinetic calculation.

**Fig. 3:**
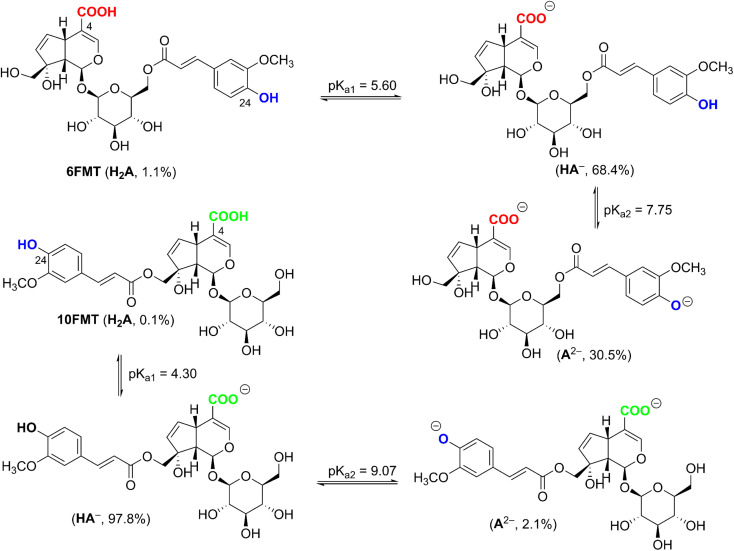
The deprotonation of FMT in water at pH = 7.4.

From the data of Table 4, the BDE values of C5–H and O24–H position of 6-FMT and 10-FMT in water are even over than that in a–polar solvent, consistent with the bond-weakening effect of the high dielectric constant of the medium. The lower BDE correspond to the lower negative Δ*G*^o^ values which range from −7.2 kcal mol^−1^ to −21.9 kcal mol^−1^. The lowest PA values of OH bonds of 6-FMT and 10-FMT were predicted for O4–H at 40.5 kcal mol^−1^ and 35.7 kcal mol^−1^, and that of O24–H at 50.0 kcal mol^−1^ and 38.7 kcal mol^−1^, respectively. Because of the lower PA value at O4–H position, both 6-FMT and 10-FMT would most likely be dissociated at O4–H follow by O24–H position. The p*K*_a_ was calculated following the literature,^50,51^ and the results are presented in Fig. 3.

The p*K*a_1_ and p*K*a_2_ of 6-FMT are 5.60 and 7.75, at pH 7.4 yielding state populations of 1.1% of neutral (HA), 68.4% of anion (HA^−^), and 30.5% of dianion (A^2−^). The p*K*a_1_ and p*K*a_2_ of 10-FMT are 4.30 and 9.07, corresponding to 0.1% of neutral, 97,8% of anion, and 2.1% of dianion populations. Thus, these populations were used for the kinetic calculation.

#### 3.2.2. Kinetic study

Based on the thermodynamic data in solvents, the HOO˙ radical scavenging reaction of 6-FMT and 10-FMT in pentyl ethanoate was modelled by the FHT mechanism at C5–H and O24–H position. Whereas the SET mechanism was investigated for dissociated state in aqueous solution, since the FHT reaction had no contribution in the HOO˙ antiradical activity of phenolic acids.^25,50,52^ The overall reaction rate constant (*k*_overall_) was calculated following the QM-ORSA protocol,^29,34^ according to eqn (2) and (3), and the results were listed in Table 5 and 6.

**Table 5:** Calculated activation Gibbs free energies (Δ*G*^‡^, kcal mol^−1^), tunneling corrections (*κ*), *k*_Eck_, *k*_overall_ (M^−1^ s^−1^) and branching ratios (*Γ*, %) for the HOO˙ scavenging of the FMT in pentyl ethanoate

Mechanism	Position	Δ*G*^‡^	*κ*	*k*_Eck_	*k*_app_	*Γ*
FHT	6-FMT–C5–H	19.8	116.6	24.0	24.0	99.6
	6-FMT–O24–H	18.9	0.1	9.4 × 10^−3^	9.4 × 10^−3^	0.4
	*k*_overall_				24.0	
FHT	10-FMT-C5-H	19.1	43.2	2.50	2.50	9.7
	10-FMT-O24–H	19.3	488.0	23.0	23.0	90.3
	*k*_overall_				25.5	

**Table 6:** Calculated activation Gibbs free energies (Δ*G*^‡^, kcal mol^−1^), tunnelling corrections (*κ*), *k*_Eck_, *k*_overall_ (M^−1^ s^−1^) and branching ratios (*Γ*, %) for the HOO˙ scavenging of the FMT in water following the SET pathway

Comp.	States	Δ*G*^‡^	*κ*	*k*_D_	*k*_app_	*f*	*k*_f_	*Γ*
6-FMT	HA^−^	29.8	17.0	8.80 × 10^9^	9.50 × 10^−10^	0.684	6.50 × 10^−10^	0.0
	A^2–^	6.4	12.9	8.80 × 10^9^	1.20 × 10^8^	0.305	3.66 × 10^7^	100.0
							3.66 × 10^7^	Chem
10-FMT	HA^−^	33.5	15.9	8.70 × 10^9^	1.70 × 10^−12^	0.978	1.66 × 10^−12^	0.0
	A^2−^	5.6	16.0	8.70 × 10^9^	4.50 × 10^8^	0.021	9.45 × 10^6^	100.0
							9.45 × 10^6^	Chem

In the pentyl ethanoate solvent:

2 *k*_overall_ = ∑*k*_app_(FHT–neutral)

In water:

3 *k*_overall_ = ∑*k*_app_(SET–anion) + ∑*k*_app_(SET–dianion)

As per calculated data, the *k*_overall_ of HOO˙ radical scavenging reaction of 6-FMT and 10-FMT in pentyl ethanoate are 24.0 M^−1^ s^−1^ and 25.5 M^−1^ s^−1^, respectively. These rate constants suggest that 6-FMT and 10-FMT are weak antioxidants in non-polar environments. In contrast, both 6-FMT and 10-FMT have good radical scavenging activity in water with the *k*_overall_ at 3.66 × 10^7^ M^−1^ s^−1^ for 6-FMT and 9.45 × 10^6^ M^−1^ s^−1^ for 10-FMT. The radical scavenging activity of FMT+HOO· reactions are dominated by the SET mechanism of A^2−^ in both cases, with *k*_*f*_(A^2−^) = 3.66 × 10^7^ M^−1^ s^−1^, *Γ* = 100% for 6-FMT and *k*_*f*_ (A^2−^) = 9.45 × 10^6^ M^−1^ s^−1^ for 10-FMT, while the HA^−^ state do not make any contribution (*Γ* = 0.0%). Those calculated rate of activity of FMTs are nearly equivalent to their moieties, such as ferulic acid (*k*_overall_ = 5.80 × 10^7^ M^−1^ s^−1^),^53^ guaiacol (*k*_overall_ = 2.38 × 10^6^ M^−1^ s^−1^).^54^ Based on calculated data, we can conclude that 6-FMT and 10-FMT are better HOO˙ radical scavengers in the aqueous solution than the reference antioxidant trolox, with the rate of activity approximately 105–408 times faster than that of trolox (*k* = 8.96 × 10^4^ M^−1^ s^−1^).^55^ The rate constants are similar to ascorbic acid (*k* = 9.97 × 10^7^ M^−1^ s^−1^)^34^ and resveratrol ((*k* = 5.62 × 10^7^ M^−1^ s^−1^),^56^ suggesting that 6-FMT and 10-FMT are good antioxidants in the polar environment.

## 4. Conclusion

In this study, the HOO˙ radical scavenging activity of 6-FMT and 10-FMT were calculated by M06-2X functional. The results show that both of the studied substances are weak antioxidant in apolar environment, but they show good antioxidant activity in the polar environment, with *k*_overall_ = 3.66 × 10^7^ M^−1^ s^−1^ for 6-FMT and 9.45 × 10^6^ M^−1^ s^−1^ for 10-FMT, *via* SET pathway at O4–H and O24–H position. The activity is exerted by the dianion A^2−^ with *Γ* = 100% while the anion HA^−^ does not make any contribution. These rate constants of radical scavenging activity are substantially better than reference antioxidant trolox and similar to ascorbic acid and resveratrol, confirming that 6-FMT and 10-FMT are good antioxidants in water at physiological pH.

## Conflicts of interest

There are no conflicts to declare.

## Supplementary Material

RA-013-D3RA00458A-s001
