# Supplementary material for: Feruloylmonotropeins: promising natural antioxidants in Paederia scandens
Source: RSC Adv. 2023 Feb 20;13(9):6153–9. doi: 10.1039/d3ra00458a (PMC9940704; doi:10.1039/d3ra00458a)
Supplement: RA-013-D3RA00458A-s001 [file RA-013-D3RA00458A-s001.pdf]

Electronic Supplementary Material (ESI) for RSC Advances.
This journal is © The Royal Society of Chemistry 2023

Supporting Information (SI)

Feruloylmonotropeins: promising natural antioxidants in *Paederia scandens*

Nguyen Quang Trung,[1,2*] Nguyen Thi Thu Thanh,[3] Nguyen Thi Hoa,[3] Adam Mechler,[5] Quan V. Vo[4*]

[1]The University of Danang – University of Science and Education, Da Nang 550000, Vietnam.

[2]Quality assurance and Testing center 2, Da Nang 550000, Vietnam.

[3]Le Thanh Phuong High School, An My, Phu Yen 621640, Vietnam

[4]The University of Danang – University of Technology and Education, Danang 550000, Vietnam.

[5]Department of Biochemistry and Chemistry, La Trobe University, Victoria 3086, Australia.

*Corresponding authors: nqtrung.quatest2@gmail.com; vvquan@ute.udn.vn;

Table of Contents

Table S1. The method to calculate rate constant following the conventional transition state theory S2

Table S2: The Cartesian coordinates and energies of FMT, ANION, DIANION, TSs in the studied media (G: gas phase; P: pentyl ethanoate; H: water) S3

References S25

Table S1. The method to calculate rate constant following the conventional transition state theory

The rate constant (k) was calculated by using the conventional transition state theory (TST) (at 298.15 K, 1M standard state) according to the equation (1):[1-5]

$$k = \sigma\kappa \frac{k_B T}{h} e^{-(\Delta G^{\neq})/RT} \quad (1)$$

Where: σ is the reaction symmetry number ,[6,7]
κ contains the tunneling corrections calculated using the Eckart barrier,[8]
k_B is the Boltzmann constant,
h is the Planck constant,
$\Delta G^{\neq}$ is the Gibbs free energy of activation.

The Marcus Theory was used to estimate the reaction barriers of SET reactions.[9-12] The free energy of reaction $\Delta G^{\neq}$ for the SET pathway was computed following the equations (2,3).

$$\Delta G^{\neq}_{SET} = \frac{\lambda}{4}\left(1 + \frac{\Delta G^0_{SET}}{\lambda}\right)^2 \quad (2)$$

$$\lambda \approx \Delta E_{SET} - \Delta G^0_{SET} \quad (3)$$

where ΔG_{SET} is the Gibbs energy of reaction, ΔE_{SET} is the non-adiabatic energy difference between reactants and vertical products for SET.[13,14]

For rate constants that were close to the diffusion limit a correction was applied to yield realistic results[15]. The apparent rate constants (k_{app}) were calculated following the Collins–Kimball theory in the solvents at 298.15K;[16] the steady-state Smoluchowski rate constant (k_D) for an irreversible bimolecular diffusion–controlled reaction was calculated following the literature as corroding to equations (4,5).[15,17]

$$k_{app} = \frac{k_{TST} k_D}{k_{TST} + k_D} \quad (4)$$

$$k_D = 4\pi R_{AB} D_{AB} N_A \quad (5)$$

where R_{AB} is the reaction distance, N_A is the Avogadro constant, and $D_{AB} = D_A + D_B$ (D_{AB} is the mutual diffusion coefficient of the reactants A and B),[16,18] where D_A or D_B is estimated using the Stokes–Einstein formulation (6).[19,20]

$$D_{A\ or\ B} = \frac{k_B T}{6\pi\eta a_{A\ or\ B}} \quad (6)$$

η is the viscosity of the solvents (i.e. $\eta(H_2O) = 8.91\times10^{-4}$ Pa s, η(pentyl ethanoate) = 8.62×10^{-4} Pa s) and a is the radius of the solute.

The kinetic study requires different considerations. Water and pentyl ethanoate are the *de facto* standard solvents in the literature to mimic the polar and nonpolar environments in the human body.[15,21-23] Thus, these solvents were used to model the physiological environments. The solvent cage effects were included following the corrections proposed by Okuno,[24] adjusted with the free volume theory according to the Benson correction[15,25-27] to reduce over-penalizing entropy losses in solution. For the species that have multiple conformers, all of these were investigated and the conformer with the lowest electronic energy was included in the analysis.[22,23] The hindered internal rotation treatment was also applied to the single bonds to ensure that the obtained conformer has the lowest electronic energy.[23,28] All transition states were characterized by the existence of only one single imaginary frequency. Intrinsic coordinate calculations (IRCs) were performed to ensure that each transition state is connected correctly with the pre-complex and post-complex. The kinetic calculations were performed by the *Eyringpy* code[29,30]

Table S2: The Cartesian coordinates and energies of FMT, ANION, DIANION, TSs in the studied media (G: gas phase; P: pentyl ethanoate; H: water)

**Name**	**6-FMT-G**
Cartesian Coordinates	Energy
C 3.23396500 -1.79783900 0.63228400<br>C 1.81284900 -2.33312700 0.29583200<br>C 2.08366000 -3.76379500 -0.13015200<br>C 3.38426900 -4.03953400 -0.22351400<br>C 4.25453400 -2.85987600 0.13068000<br>C 3.46281300 -0.41671000 0.04954000<br>O 3.12949100 -0.37268400 -1.34608300<br>C 1.84842900 -0.72907800 -1.60286900<br>C 1.14999600 -1.56421700 -0.82417200<br>O 2.64080700 0.45427000 0.77736700<br>C 2.32984800 2.66930600 1.51957800<br>C 2.52567700 1.76001500 0.31272000<br>O 1.38859200 1.80136600 -0.53918000<br>C 1.05754200 3.09639300 -1.01918800<br>C 0.71195700 4.00214000 0.16701300<br>C 1.93171600 4.06459000 1.06855400<br>O 3.54304900 2.69184400 2.23287600<br>O 1.69434300 4.82206600 2.23442400<br>O 0.41721500 5.32017600 -0.23047800<br>C -0.06200200 2.92765200 -2.02963800<br>O -1.19711700 2.27126100 -1.45869400<br>C 5.30056800 -3.17874100 1.19854700<br>O 5.04375600 -2.42567500 -0.97913800<br>O 6.23659300 -4.12008100 0.73369100<br>C -2.18217100 3.05725000 -0.94855100<br>C -3.32973900 2.31902100 -0.42085100<br>C -3.40409300 0.97556600 -0.47390100<br>C -4.45466300 0.10383100 0.03347500<br>O -2.09003800 4.27287600 -0.93169500<br>C -5.66378600 0.56614700 0.56441200<br>C -6.61493200 -0.33067900 1.03818000<br>C -6.36500700 -1.69786800 0.98929300<br>C -5.15144200 -2.17513200 0.45815300<br>C -4.20682800 -1.28341500 -0.02003400<br>O -7.28781100 -2.57071100 1.45039100<br>O -5.03546800 -3.53259500 0.47163000<br>C -3.81542300 -4.08200300 -0.00821700<br>C -0.28920900 -1.77260200 -1.07591100<br>O -0.89562900 -2.77561700 -0.77699900<br>O -0.94633300 -0.74009700 -1.65923600<br>H 3.32985200 -1.69814800 1.71906800<br>H 1.16043700 -2.30551700 1.17557000<br>H 1.26563100 -4.44292200 -0.34886800<br>H 3.82205500 -4.98622500 -0.52486000<br>H 4.50878300 -0.09882600 0.07865600<br>H 1.46084900 -0.25344900 -2.49889400<br>H 1.51823400 2.24430100 2.13001700<br>H 3.42478700 2.05760500 -0.25349800<br>H 1.91452100 3.53878700 -1.55715600	Zero-point correction= 0.564368 (Hartree/Particle)<br>Thermal correction to Energy= 0.601146<br>Thermal correction to Enthalpy= 0.602090<br>Thermal correction to Gibbs Free Energy= 0.494663<br>Sum of electronic and zero-point Energies= -2060.607379<br>Sum of electronic and thermal Energies= -2060.570601<br>Sum of electronic and thermal Enthalpies= -2060.569656<br>Sum of electronic and thermal Free Energies= -2060.677083

H -0.12314100 3.56042800 0.73115400	
H 2.76547000 4.50757500 0.49640900	
H 3.46289200 3.37485600 2.91765300	
H 1.33642400 5.68035100 1.95517900	
H -0.51504800 5.33655500 -0.51518700	
H 0.27242600 2.27266300 -2.83887000	
H -0.35146400 3.89633100 -2.44356800	
H 5.79378700 -2.23965500 1.49848500	
H 4.81366200 -3.61338400 2.07597800	
H 4.46697500 -2.24169900 -1.73639400	
H 6.57132800 -3.78786000 -0.11468300	
H -4.09109900 2.94983900 0.02576600	
H -2.58233400 0.44522600 -0.94320900	
H -5.87237400 1.63101200 0.60289500	
H -7.56043700 0.00757700 1.44937900	
H -3.26618300 -1.64065600 -0.43352700	
H -6.93843000 -3.47092200 1.33475000	
H -3.91258100 -5.16188400 0.09699500	
H -3.65726000 -3.82023700 -1.05947300	
H -2.96696000 -3.71889200 0.57970000	
H -0.47357200 0.10221200 -1.51805000	
**Name**	**10-FMT-G**
Cartesian Coordinates	Energy
C -2.78418700 0.56122800 -0.53206600	Zero-point correction= 0.565120 (Hartree/Particle)
C -4.11214400 -0.00116600 -1.09802800	Thermal correction to Energy= 0.601579
C -5.16963700 0.89948200 -0.49936200	Thermal correction to Enthalpy= 0.602523
C -4.63985100 1.97579800 0.07600400	Thermal correction to Gibbs Free Energy= 0.497076
C -3.12749200 1.96487800 0.07483600	Sum of electronic and zero-point Energies= -2060.611523
C -2.17279600 -0.31823500 0.54534700	Sum of electronic and thermal Energies= -2060.575063
O -2.10527400 -1.69633700 0.13456700	Sum of electronic and thermal Enthalpies= -2060.574119
C -3.23988400 -2.21539200 -0.36421700	Sum of electronic and thermal Free Energies= -2060.679566
C -4.24623800 -1.48616300 -0.87479900	
O -0.86060500 0.09499800 0.74353800	
C 0.94250900 0.74777900 2.15796100	
C -0.36998000 -0.05072800 2.07652300	
O -0.26891200 -1.39109600 2.45282400	
C 0.90356700 -2.17239300 2.17516000	
C 2.18630700 -1.33770600 2.13242300	
C 1.98707900 -0.05363100 2.91041800	
O 0.78397900 1.98364700 2.82358700	
O 3.22520000 0.62330700 2.93434400	
O 3.22672900 -2.13671800 2.65748000	
C 0.72121000 -3.02325600 0.92638100	
O 0.71783200 -2.26363300 -0.26722700	
C -2.55403200 3.07133600 -0.81718000	
O -2.70848400 2.11215500 1.42239000	
O -1.17662000 2.83438100 -1.17139900	
C -0.22505000 3.14523500 -0.29142100	
C 1.13655200 2.79756200 -0.71296600	
C 1.40508200 1.66148300 -1.37948700	
O -0.46856200 3.65544700 0.79828800	
C 2.75477200 1.16651900 -1.65714900	
C 3.81266300 2.03861200 -1.92902900	
C 5.09477500 1.54667100 -2.15778500	

C 5.33078200 0.17993500 -2.09344500<br>C 4.27259000 -0.70503200 -1.80876300<br>C 2.98914600 -0.22164700 -1.61005000<br>O 6.57515900 -0.30414600 -2.30628900<br>H -2.02982100 0.64132900 -1.32103300<br>H -4.12498300 0.15163000 -2.18808300<br>H -6.22863200 0.68720200 -0.60139400<br>H -5.18288600 2.78405900 0.55633300<br>H -2.75026200 -0.29366900 1.47693800<br>H -3.26444600 -3.30054700 -0.33392400<br>H 1.32047700 0.90497700 1.13990500<br>H -1.10672900 0.38686200 2.75906200<br>H 0.99100100 -2.84313300 3.03788300<br>H 2.42192500 -1.05626800 1.09368500<br>H 1.64566000 -0.27043100 3.93453500<br>H 0.47176800 2.66420600 2.20038800<br>H 3.05637200 1.52457500 3.25329100<br>H 4.00048500 -1.55902100 2.76220600<br>H -0.20386900 -3.60612200 1.02034300<br>H 1.56523700 -3.71932300 0.86088000<br>H -0.12806900 -1.78674500 -0.29908600<br>H -3.07469200 3.07824200 -1.77650600<br>H -2.64602800 4.05069100 -0.33715200<br>H -1.95394500 2.72445100 1.47116800<br>H 1.92248000 3.40418900 -0.27147800<br>H 0.57264200 1.01641600 -1.66168300<br>H 3.62789200 3.10708700 -1.99330800<br>H 5.92475200 2.20707200 -2.38681600<br>H 2.17336000 -0.90220000 -1.37450100<br>H 6.54590600 -1.27034100 -2.19984600<br>C -5.40215500 -2.24309200 -1.38781800<br>O -5.59005000 -3.43283200 -1.26577900<br>O -6.27468200 -1.44600200 -2.05020000<br>H -6.99453200 -2.01935500 -2.36641000<br>O 4.64822800 -2.01483800 -1.76368800<br>C 3.70785300 -2.94301800 -1.24074500<br>H 2.80514200 -2.98376800 -1.85799500<br>H 4.20917100 -3.91040500 -1.24598600<br>H 3.43447500 -2.66866400 -0.21537400	
**Name**	**6-FMT-C5-H-OOH-G**
Cartesian Coordinates	Energy
C 3.14031100 -1.53118100 0.27097900<br>C 1.80964400 -2.09040500 -0.25030200<br>C 2.10719700 -3.48729200 -0.65776200<br>C 3.41762400 -3.76120800 -0.56959400<br>C 4.22606500 -2.55448700 -0.15038100<br>C 3.36724700 -0.10521100 -0.19813800<br>O 3.03773000 0.05965500 -1.58806500<br>C 1.76611000 -0.28192600 -1.88602900<br>C 1.09046900 -1.24097600 -1.22918700<br>O 2.53222300 0.69021100 0.59441100<br>C 2.17473800 2.82836700 1.51192300<br>C 2.37881100 2.02522200 0.23373700<br>O 1.23112900 2.09503700 -0.60207300	Zero-point correction= 0.575080 (Hartree/Particle)<br>Thermal correction to Energy= 0.615289<br>Thermal correction to Enthalpy= 0.616234<br>Thermal correction to Gibbs Free Energy= 0.500323<br>Sum of electronic and zero-point Energies= -2211.414574<br>Sum of electronic and thermal Energies= -2211.374364<br>Sum of electronic and thermal Enthalpies= -2211.373420<br>Sum of electronic and thermal Free Energies= -2211.489331

C	0.83083700	3.40946000	-0.96107500	
C	0.47320300	4.19399100	0.30532100	
C	1.71033200	4.23732600	1.18374900	
O	3.40537800	2.84418400	2.19515000	
O	1.46865700	4.88792300	2.41127900	
O	0.10898800	5.52517000	0.02647900	
C	-0.30495600	3.27310600	-1.95884300	
O	-1.39160600	2.51252600	-1.42358700	
C	5.21359700	-2.84121800	0.97643900	
O	5.05187100	-2.08993900	-1.22157500	
O	6.18971400	-3.77186500	0.57286200	
C	-2.39667900	3.20185200	-0.81888800	
C	-3.48456000	2.36454100	-0.31396500	
C	-3.49413600	1.02746800	-0.47207100	
C	-4.47161300	0.06225200	0.01035800	
O	-2.36387200	4.41514500	-0.70538900	
C	-5.67867700	0.41325600	0.62456400	
C	-6.55181600	-0.56995400	1.07651900	
C	-6.22361100	-1.91295700	0.92516800	
C	-5.01130700	-2.27836900	0.30937000	
C	-4.14705700	-1.30032300	-0.15083800	
O	-7.06898300	-2.86963300	1.36792000	
O	-4.81202200	-3.62371200	0.22852500	
C	-3.57593400	-4.06132800	-0.32050200	
C	-0.33113200	-1.49873000	-1.53978400	
O	-0.87115300	-2.57064900	-1.40011900	
O	-1.03871700	-0.43626900	-1.99049300	
H	3.10149700	-1.49731700	1.36799800	
H	1.32613800	-4.15982100	-0.99893000	
H	3.89919100	-4.70244500	-0.81706300	
H	4.41090700	0.21612500	-0.13800300	
H	1.36508900	0.28771100	-2.71922000	
H	1.39759700	2.32136200	2.10388500	
H	3.26262200	2.39159300	-0.31579900	
H	1.65283400	3.93681800	-1.47657700	
H	-0.32677900	3.66735900	0.84680800	
H	2.51159400	4.76012300	0.63286600	
H	3.30850400	3.44286400	2.95259900	
H	1.06411900	5.74724600	2.20975300	
H	-0.83067600	5.52154200	-0.23286800	
H	0.03925200	2.70946200	-2.83045600	
H	-0.65183200	4.25824600	-2.27903700	
H	5.67253900	-1.89147400	1.29439700	
H	4.68294800	-3.27286600	1.82925000	
H	4.51181400	-1.96153400	-2.01691400	
H	6.59159600	-3.42731000	-0.24041100	
H	-4.25563800	2.91606800	0.21356400	
H	-2.67009100	0.58087500	-1.01769600	
H	-5.94628200	1.45847400	0.74640200	
H	-7.49430400	-0.31861400	1.55189900	
H	-3.21115000	-1.57149300	-0.63396800	
H	-6.66929100	-3.73668300	1.18273000	
H	-3.59986000	-5.14968400	-0.27817100	
H	-3.46970600	-3.72860300	-1.35785900	
H	-2.73381400	-3.67860900	0.26526700	

H -0.62033000 0.40743300 -1.73153700<br>O 1.72566000 -3.19391000 2.48232500<br>H 1.53293200 -4.12654400 2.27912800<br>O 0.65451200 -2.51224800 1.93882500<br>H 1.06428700 -2.22096800 0.81157100	
**Name**	**6-FMT-O24-H-OOH-G**
Cartesian Coordinates	Energy
C 3.16544700 -2.24018100 0.59218700<br>C 1.70564400 -2.49399100 0.11900700<br>C 1.75794700 -3.92441400 -0.38384700<br>C 2.99364800 -4.42348800 -0.40017500<br>C 4.02335300 -3.44048300 0.09765800<br>C 3.67878900 -0.89414500 0.11901400<br>O 3.47503800 -0.71863600 -1.29156400<br>C 2.17744500 -0.82375100 -1.66121300<br>C 1.28153700 -1.55790200 -0.98963200<br>O 2.96097800 0.07040700 0.83945400<br>C 2.99510600 2.25881100 1.71011100<br>C 3.11341000 1.39853700 0.45813400<br>O 2.06546800 1.69326800 -0.45836000<br>C 2.00336400 3.05276700 -0.86407700<br>C 1.73772700 3.93613800 0.35932900<br>C 2.88206400 3.72683500 1.33469300<br>O 4.13976500 2.02011600 2.49287700<br>O 2.69757700 4.44411800 2.53449300<br>O 1.70810600 5.30597000 0.03545600<br>C 0.94939700 3.14972900 -1.95139200<br>O -0.32153500 2.66646000 -1.50188600<br>C 4.90077800 -3.99969900 1.21742500<br>O 4.96896500 -3.09614500 -0.91713400<br>O 5.69057700 -5.07054700 0.76261700<br>C -1.19024600 3.57299900 -0.99163500<br>C -2.48553800 3.00651500 -0.59496900<br>C -2.78393400 1.70627400 -0.77209200<br>C -4.00528300 1.01021700 -0.39013200<br>O -0.90372300 4.74978700 -0.86762400<br>C -5.14349500 1.66528400 0.12757200<br>C -6.25717700 0.93985000 0.48350500<br>C -6.29891700 -0.46797600 0.32044600<br>C -5.12909400 -1.12661500 -0.18327600<br>C -4.00928500 -0.38634400 -0.54086000<br>O -7.39447900 -1.12852600 0.56608500<br>O -5.21100300 -2.47421100 -0.26080000<br>C -4.15646100 -3.16495000 -0.93085100<br>C -0.14661000 -1.47839400 -1.34824000<br>O -0.95055300 -2.36280600 -1.16034800<br>O -0.55594600 -0.30727200 -1.89684000<br>H 3.18639400 -2.21488000 1.68721900<br>H 0.99779100 -2.39882100 0.94976900<br>H 0.85553600 -4.43389300 -0.70700300<br>H 3.28315200 -5.41598400 -0.73154400<br>H 4.75769700 -0.76903200 0.24527100<br>H 1.95359700 -0.23972500 -2.54911200<br>H 2.08011300 1.95294300 2.24052500	Zero-point correction= 0.575766 (Hartree/Particle)<br>Thermal correction to Energy= 0.615475<br>Thermal correction to Enthalpy= 0.616419<br>Thermal correction to Gibbs Free Energy= 0.501047<br>Sum of electronic and zero-point Energies= -2211.420636<br>Sum of electronic and thermal Energies= -2211.380927<br>Sum of electronic and thermal Enthalpies= -2211.379983<br>Sum of electronic and thermal Free Energies= -2211.495355

H 4.08901400 1.55969700 -0.03108700	
H 2.96116700 3.36480600 -1.31644000	
H 0.80054500 3.62035500 0.84157600	
H 3.81928700 4.04523700 0.84602700	
H 4.13054700 2.65951000 3.22290700	
H 2.52833100 5.37050900 2.29849600	
H 0.81420600 5.51112500 -0.29293600	
H 1.22293300 2.49954400 -2.78666100	
H 0.85951400 4.17897100 -2.30637200	
H 5.52513200 -3.18296900 1.61525200	
H 4.27096300 -4.38289700 2.02511900	
H 4.50058200 -2.78715400 -1.70768400	
H 6.15541700 -4.76046400 -0.03100000	
H -3.15656900 3.72534800 -0.13657700	
H -2.03868000 1.07240000 -1.24069000	
H -5.14460400 2.74493000 0.24078400	
H -7.15044500 1.41502500 0.87555300	
H -3.12119600 -0.87717600 -0.93255500	
H -4.46420500 -4.20928400 -0.95644900	
H -4.03896300 -2.78304200 -1.94965700	
H -3.20767800 -3.05897300 -0.39693100	
H 0.05451100 0.41802800 -1.66425000	
O -5.57296200 -2.22435500 2.49946900	
H -5.13786500 -2.69753500 1.76069500	
O -6.88531900 -2.56562700 2.36075000	
H -7.23155200 -1.95232700 1.37447900	
**Name**	**10-FMT-C5-H-OOH-G**
Cartesian Coordinates	Energy
C 2.61888400 -0.37426900 0.02942400	Zero-point correction= 0.576179 (Hartree/Particle)
C 3.96990800 0.13506300 -0.48220600	Thermal correction to Energy= 0.616040
C 5.00122600 -0.52731500 0.34363700	Thermal correction to Enthalpy= 0.616984
C 4.46469600 -1.49550600 1.09913200	Thermal correction to Gibbs Free Energy= 0.502502
C 2.95589800 -1.55993100 0.98918400	Sum of electronic and zero-point Energies= -2211.427619
C 1.84281700 0.71343500 0.74559600	Sum of electronic and thermal Energies= -2211.387759
O 1.75148100 1.90068300 -0.07660300	Sum of electronic and thermal Enthalpies= -2211.386815
C 2.87519700 2.32209500 -0.66987100	Sum of electronic and thermal Free Energies= -2211.501296
C 3.98951500 1.57198800 -0.82153400	
O 0.54064500 0.27697400 0.94124600	
C -1.37261900 -0.06671500 2.32202000	
C -0.08526700 0.75192000 2.13554700	
O -0.26378000 2.13530900 2.11351400	
C -1.43656600 2.74189800 1.54655300	
C -2.69088700 1.87152600 1.67705200	
C -2.49608500 0.84594600 2.77461900	
O -1.22984100 -1.08380200 3.29098900	
O -3.70658200 0.13335700 2.90673300	
O -3.77824200 2.73722000 1.93161600	
C -1.18801500 3.19368200 0.11464400	
O -1.07394900 2.11573700 -0.79457900	
C 2.50037500 -2.87986800 0.35837800	
O 2.43608200 -1.37605300 2.29585200	
O 1.14410000 -2.81519400 -0.12697700	
C 0.14363400 -2.95225000 0.73933000	
C -1.19622600 -2.82723600 0.15530300	

C -1.46495500 -1.95590800 -0.83216100<br>O 0.32154200 -3.13953900 1.94073200<br>C -2.81171800 -1.65727200 -1.32338500<br>C -3.80175400 -2.64151100 -1.39137500<br>C -5.08393400 -2.32651700 -1.83314700<br>C -5.38965700 -1.01978800 -2.18912800<br>C -4.40076200 -0.01997800 -2.11284900<br>C -3.11590300 -0.33501600 -1.70093000<br>O -6.63508700 -0.70556800 -2.61220600<br>H 1.99141700 -0.70353700 -0.80671100<br>H 4.08288100 -0.45635400 -1.58716100<br>H 6.05444200 -0.27554700 0.28235100<br>H 4.99834600 -2.14891900 1.78233100<br>H 2.31600900 1.01560700 1.68733600<br>H 2.80996900 3.34340400 -1.03273800<br>H -1.66253800 -0.49332500 1.35311900<br>H 0.59503200 0.55911100 2.97248000<br>H -1.60274200 3.63144300 2.16478100<br>H -2.86685800 1.31699000 0.74128400<br>H -2.23396200 1.34094100 3.72264900<br>H -0.80961700 -1.87129900 2.89951800<br>H -3.52311100 -0.65090000 3.44898000<br>H -4.54513100 2.17606300 2.13201000<br>H -0.29061800 3.82473600 0.08651500<br>H -2.04579000 3.79446400 -0.20838700<br>H -0.22234600 1.68155900 -0.62126000<br>H 3.09455700 -3.07956700 -0.53512600<br>H 2.59431100 -3.70668300 1.06964700<br>H 1.71315500 -2.00952700 2.45283300<br>H -1.98474800 -3.33245700 0.70643100<br>H -0.64347900 -1.36983800 -1.24500000<br>H -3.56107800 -3.66750100 -1.12812500<br>H -5.86026500 -3.08080900 -1.90940300<br>H -2.35530600 0.43880600 -1.62407500<br>H -6.65997600 0.24857800 -2.79801000<br>C 5.14015800 2.21860000 -1.47753800<br>O 5.21463700 3.38283700 -1.79776000<br>O 6.15297500 1.34688300 -1.70152700<br>H 6.85874600 1.84955500 -2.14467400<br>O -4.83968900 1.21803400 -2.47948500<br>C -3.97368600 2.31438700 -2.22506100<br>H -3.04041000 2.22137300 -2.78934300<br>H -4.51632000 3.20416200 -2.54278000<br>H -3.74388500 2.37632700 -1.15534600<br>O 4.80813800 -2.35922800 -2.10245900<br>H 5.72364700 -2.15099200 -2.36225900<br>O 4.07882500 -1.30198000 -2.58399800	
**Name**	**10-FMT-O24-H-OOH-G**
Cartesian Coordinates	Energy
C 3.06174600 -0.03735000 -0.86602500<br>C 4.25279000 0.89308000 -1.20521900<br>C 5.43998300 -0.04294100 -1.24958700<br>C 5.06874700 -1.32053800 -1.23854300<br>C 3.57636200 -1.50302600 -1.07529000	Zero-point correction= 0.576618 (Hartree/Particle)<br>Thermal correction to Energy= 0.615889<br>Thermal correction to Enthalpy= 0.616834<br>Thermal correction to Gibbs Free Energy= 0.504160<br>Sum of electronic and zero-point Energies= -2211.424833

C	2.56645100	0.12282500	0.56071400	Sum of electronic and thermal Energies=	-2211.385562
O	2.34720200	1.50564300	0.89502400	Sum of electronic and thermal Enthalpies=	-2211.384618
C	3.36517700	2.34039100	0.61708000	Sum of electronic and thermal Free Energies=	-2211.497292
C	4.32867000	2.08357100	-0.28282800		
O	1.33239500	-0.50845000	0.65770500		
C	-0.16844700	-2.01632700	1.72996400		
C	1.03319300	-1.07499200	1.93383900		
O	0.85880000	-0.09550200	2.91253100		
C	-0.40693400	0.54678400	3.12765200		
C	-1.59592800	-0.32929000	2.73037000		
C	-1.20023400	-1.78894100	2.81740500		
O	0.20271600	-3.37903800	1.74917800		
O	-2.36145700	-2.55594000	2.58194600		
O	-2.66843000	0.00015200	3.58822200		
C	-0.45064800	1.92027800	2.47307900		
O	-0.51679100	1.86117700	1.06046400		
C	2.93730500	-2.10557100	-2.33136900		
O	3.38472700	-2.33699400	0.05644100		
O	1.51124200	-1.88661800	-2.38449800		
C	0.70979200	-2.68023900	-1.67939100		
C	-0.71777700	-2.33029000	-1.76595800		
C	-1.13450200	-1.05394500	-1.72955200		
O	1.11938600	-3.61157700	-0.99579700		
C	-2.53868100	-0.65495900	-1.61160000		
C	-3.56618100	-1.40963000	-2.21540600		
C	-4.88203600	-1.02914100	-2.07291300		
C	-5.23585800	0.08957700	-1.27902600		
C	-4.18131600	0.85116200	-0.67693400		
C	-2.85198300	0.48689100	-0.86016800		
O	-6.48790100	0.37418800	-1.05950900		
H	2.20466200	0.18180500	-1.51038100		
H	4.11029300	1.29523000	-2.21992300		
H	6.45668600	0.32420500	-1.34125000		
H	5.72554900	-2.18376400	-1.28596400		
H	3.28112300	-0.27054700	1.29328000		
H	3.32848200	3.26570200	1.18397200		
H	-0.64691000	-1.76542600	0.77587600		
H	1.90119700	-1.65825700	2.25998800		
H	-0.46262900	0.68366900	4.21415000		
H	-1.88192200	-0.12794100	1.68540700		
H	-0.77890000	-2.01829400	3.80833400		
H	0.43811700	-3.66306900	0.84841900		
H	-2.08604200	-3.48161200	2.48573600		
H	-3.35336900	-0.67707300	3.46007600		
H	0.42188100	2.50091500	2.79807500		
H	-1.35579800	2.43429200	2.81619100		
H	0.36594900	1.60486300	0.74507200		
H	3.31227500	-1.59420600	-3.21959300		
H	3.14512700	-3.17776000	-2.40283600		
H	2.65746500	-2.96096300	-0.10886300		
H	-1.40402200	-3.16561300	-1.65556800		
H	-0.38844800	-0.26034100	-1.69117600		
H	-3.31043000	-2.27233800	-2.82377200		
H	-5.69262800	-1.58009000	-2.53852200		
H	-2.05331700	1.05610100	-0.38846700		

C 5.35160800 3.13172200 -0.45628000	
O 5.48370800 4.12100000 0.22823400	
O 6.16273200 2.87603700 -1.51017100	
H 6.79467800 3.61408700 -1.56501100	
O -4.58108900 1.92776700 0.04091300	
C -3.66693500 2.46379000 0.99905100	
H -2.78421100 2.89421500 0.51647700	
H -4.22315200 3.23637300 1.52879900	
H -3.36076700 1.67991200 1.70068100	
O -5.57214700 2.74670300 -2.41413900	
H -5.10547300 2.97852800 -1.58431600	
O -6.84839600 2.50185300 -1.99977400	
H -6.75522000 1.46683900 -1.39743700	
**Name**	**6-FMT-P**
Cartesian Coordinates	Energy
C 3.23396500 -1.79783900 0.63228400	Zero-point correction= 0.562421 (Hartree/Particle)
C 1.81284900 -2.33312700 0.29583200	Thermal correction to Energy= 0.598826
C 2.08366000 -3.76379500 -0.13015200	Thermal correction to Enthalpy= 0.599770
C 3.38426900 -4.03953400 -0.22351400	Thermal correction to Gibbs Free Energy= 0.493731
C 4.25453400 -2.85987600 0.13068000	Sum of electronic and zero-point Energies= -2060.647747
C 3.46281300 -0.41671000 0.04954000	Sum of electronic and thermal Energies= -2060.611342
O 3.12949100 -0.37268400 -1.34608300	Sum of electronic and thermal Enthalpies= -2060.610398
C 1.84842900 -0.72907800 -1.60286900	Sum of electronic and thermal Free Energies= -2060.716437
C 1.14999600 -1.56421700 -0.82417200	
O 2.64080700 0.45427000 0.77736700	
C 2.32984800 2.66930600 1.51957800	
C 2.52567700 1.76001500 0.31272000	
O 1.38859200 1.80136600 -0.53918000	
C 1.05754200 3.09639300 -1.01918800	
C 0.71195700 4.00214000 0.16701300	
C 1.93171600 4.06459000 1.06855400	
O 3.54304900 2.69184400 2.23287600	
O 1.69434300 4.82206600 2.23442400	
O 0.41721500 5.32017600 -0.23047800	
C -0.06200200 2.92765200 -2.02963800	
O -1.19711700 2.27126100 -1.45869400	
C 5.30056800 -3.17874100 1.19854700	
O 5.04375600 -2.42567500 -0.97913800	
O 6.23659300 -4.12008100 0.73369100	
C -2.18217100 3.05725000 -0.94855100	
C -3.32973900 2.31902100 -0.42085100	
C -3.40409300 0.97556600 -0.47390100	
C -4.45466300 0.10383100 0.03347500	
O -2.09003800 4.27287600 -0.93169500	
C -5.66378600 0.56614700 0.56441200	
C -6.61493200 -0.33067900 1.03818000	
C -6.36500700 -1.69786800 0.98929300	
C -5.15144200 -2.17513200 0.45815300	
C -4.20682800 -1.28341500 -0.02003400	
O -7.28781100 -2.57071100 1.45039100	
O -5.03546800 -3.53259500 0.47163000	
C -3.81542300 -4.08200300 -0.00821700	
C -0.28920900 -1.77260200 -1.07591100	
O -0.89562900 -2.77561700 -0.77699900	

O	-0.94633300	-0.74009700	-1.65923600	
H	3.32985200	-1.69814800	1.71906800	
H	1.16043700	-2.30551700	1.17557000	
H	1.26563100	-4.44292200	-0.34886800	
H	3.82205500	-4.98622500	-0.52486000	
H	4.50878300	-0.09882600	0.07865600	
H	1.46084900	-0.25344900	-2.49889400	
H	1.51823400	2.24430100	2.13001700	
H	3.42478700	2.05760500	-0.25349800	
H	1.91452100	3.53878700	-1.55715600	
H	-0.12314100	3.56042800	0.73115400	
H	2.76547000	4.50757500	0.49640900	
H	3.46289200	3.37485600	2.91765300	
H	1.33642400	5.68035100	1.95517900	
H	-0.51504800	5.33655500	-0.51518700	
H	0.27242600	2.27266300	-2.83887000	
H	-0.35146400	3.89633100	-2.44356800	
H	5.79378700	-2.23965500	1.49848500	
H	4.81366200	-3.61338400	2.07597800	
H	4.46697500	-2.24169900	-1.73639400	
H	6.57132800	-3.78786000	-0.11468300	
H	-4.09109900	2.94983900	0.02576600	
H	-2.58233400	0.44522600	-0.94320900	
H	-5.87237400	1.63101200	0.60289500	
H	-7.56043700	0.00757700	1.44937900	
H	-3.26618300	-1.64065600	-0.43352700	
H	-6.93843000	-3.47092200	1.33475000	
H	-3.91258100	-5.16188400	0.09699500	
H	-3.65726000	-3.82023700	-1.05947300	
H	-2.96696000	-3.71889200	0.57970000	
H	-0.47357200	0.10221200	-1.51805000	
**Name**				**10-FMT-P**
Cartesian Coordinates				Energy
C	-2.78418700	0.56122800	-0.53206600	Zero-point correction= 0.563269 (Hartree/Particle)
C	-4.11214400	-0.00116600	-1.09802800	Thermal correction to Energy= 0.600232
C	-5.16963700	0.89948200	-0.49936200	Thermal correction to Enthalpy= 0.601176
C	-4.63985100	1.97579800	0.07600400	Thermal correction to Gibbs Free Energy= 0.493852
C	-3.12749200	1.96487800	0.07483600	Sum of electronic and zero-point Energies= -2060.649212
C	-2.17279600	-0.31823500	0.54534700	Sum of electronic and thermal Energies= -2060.612249
O	-2.10527400	-1.69633700	0.13456700	Sum of electronic and thermal Enthalpies= -2060.611304
C	-3.23988400	-2.21539200	-0.36421700	Sum of electronic and thermal Free Energies= -2060.718628
C	-4.24623800	-1.48616300	-0.87479900	
O	-0.86060500	0.09499800	0.74353800	
C	0.94250900	0.74777900	2.15796100	
C	-0.36998000	-0.05072800	2.07652300	
O	-0.26891200	-1.39109600	2.45282400	
C	0.90356700	-2.17239300	2.17516000	
C	2.18630700	-1.33770600	2.13242300	
C	1.98707900	-0.05363100	2.91041800	
O	0.78397900	1.98364700	2.82358700	
O	3.22520000	0.62330700	2.93434400	
O	3.22672900	-2.13671800	2.65748000	
C	0.72121000	-3.02325600	0.92638100	
O	0.71783200	-2.26363300	-0.26722700	

C -2.55403200 3.07133600 -0.81718000<br>O -2.70848400 2.11215500 1.42239000<br>O -1.17662000 2.83438100 -1.17139900<br>C -0.22505000 3.14523500 -0.29142100<br>C 1.13655200 2.79756200 -0.71296600<br>C 1.40508200 1.66148300 -1.37948700<br>O -0.46856200 3.65544700 0.79828800<br>C 2.75477200 1.16651900 -1.65714900<br>C 3.81266300 2.03861200 -1.92902900<br>C 5.09477500 1.54667100 -2.15778500<br>C 5.33078200 0.17993500 -2.09344500<br>C 4.27259000 -0.70503200 -1.80876300<br>C 2.98914600 -0.22164700 -1.61005000<br>O 6.57515900 -0.30414600 -2.30628900<br>H -2.02982100 0.64132900 -1.32103300<br>H -4.12498300 0.15163000 -2.18808300<br>H -6.22863200 0.68720200 -0.60139400<br>H -5.18288600 2.78405900 0.55633300<br>H -2.75026200 -0.29366900 1.47693800<br>H -3.26444600 -3.30054700 -0.33392400<br>H 1.32047700 0.90497700 1.13990500<br>H -1.10672900 0.38686200 2.75906200<br>H 0.99100100 -2.84313300 3.03788300<br>H 2.42192500 -1.05626800 1.09368500<br>H 1.64566000 -0.27043100 3.93453500<br>H 0.47176800 2.66420600 2.20038800<br>H 3.05637200 1.52457500 3.25329100<br>H 4.00048500 -1.55902100 2.76220600<br>H -0.20386900 -3.60612200 1.02034300<br>H 1.56523700 -3.71932300 0.86088000<br>H -0.12806900 -1.78674500 -0.29908600<br>H -3.07469200 3.07824200 -1.77650600<br>H -2.64602800 4.05069100 -0.33715200<br>H -1.95394500 2.72445100 1.47116800<br>H 1.92248000 3.40418900 -0.27147800<br>H 0.57264200 1.01641600 -1.66168300<br>H 3.62789200 3.10708700 -1.99330800<br>H 5.92475200 2.20707200 -2.38681600<br>H 2.17336000 -0.90220000 -1.37450100<br>H 6.54590600 -1.27034100 -2.19984600<br>C -5.40215500 -2.24309200 -1.38781800<br>O -5.59005000 -3.43283200 -1.26577900<br>O -6.27468200 -1.44600200 -2.05020000<br>H -6.99453200 -2.01935500 -2.36641000<br>O 4.64822800 -2.01483800 -1.76368800<br>C 3.70785300 -2.94301800 -1.24074500<br>H 2.80514200 -2.98376800 -1.85799500<br>H 4.20917100 -3.91040500 -1.24598600<br>H 3.43447500 -2.66866400 -0.21537400	
**Name**	**6-FMT-C5-H-OOH-P**
Cartesian Coordinates	Energy
C 3.14031100 -1.53118100 0.27097900<br>C 1.80964400 -2.09040500 -0.25030200<br>C 2.10719700 -3.48729200 -0.65776200	Zero-point correction= 0.572861 (Hartree/Particle)<br>Thermal correction to Energy= 0.613782<br>Thermal correction to Enthalpy= 0.614726

C	3.41762400	-3.76120800	-0.56959400	Thermal correction to Gibbs Free Energy= 0.496221
C	4.22606500	-2.55448700	-0.15038100	Sum of electronic and zero-point Energies= -2211.461610
C	3.36724700	-0.10521100	-0.19813800	Sum of electronic and thermal Energies= -2211.420689
O	3.03773000	0.05965500	-1.58806500	Sum of electronic and thermal Enthalpies= -2211.419745
C	1.76611000	-0.28192600	-1.88602900	Sum of electronic and thermal Free Energies= -2211.538250
C	1.09046900	-1.24097600	-1.22918700	
O	2.53222300	0.69021100	0.59441100	
C	2.17473800	2.82836700	1.51192300	
C	2.37881100	2.02522200	0.23373700	
O	1.23112900	2.09503700	-0.60207300	
C	0.83083700	3.40946000	-0.96107500	
C	0.47320300	4.19399100	0.30532100	
C	1.71033200	4.23732600	1.18374900	
O	3.40537800	2.84418400	2.19515000	
O	1.46865700	4.88792300	2.41127900	
O	0.10898800	5.52517000	0.02647900	
C	-0.30495600	3.27310600	-1.95884300	
O	-1.39160600	2.51252600	-1.42358700	
C	5.21359700	-2.84121800	0.97643900	
O	5.05187100	-2.08993900	-1.22157500	
O	6.18971400	-3.77186500	0.57286200	
C	-2.39667900	3.20185200	-0.81888800	
C	-3.48456000	2.36454100	-0.31396500	
C	-3.49413600	1.02746800	-0.47207100	
C	-4.47161300	0.06225200	0.01035800	
O	-2.36387200	4.41514500	-0.70538900	
C	-5.67867700	0.41325600	0.62456400	
C	-6.55181600	-0.56995400	1.07651900	
C	-6.22361100	-1.91295700	0.92516800	
C	-5.01130700	-2.27836900	0.30937000	
C	-4.14705700	-1.30032300	-0.15083800	
O	-7.06898300	-2.86963300	1.36792000	
O	-4.81202200	-3.62371200	0.22852500	
C	-3.57593400	-4.06132800	-0.32050200	
C	-0.33113200	-1.49873000	-1.53978400	
O	-0.87115300	-2.57064900	-1.40011900	
O	-1.03871700	-0.43626900	-1.99049300	
H	3.10149700	-1.49731700	1.36799800	
H	1.32613800	-4.15982100	-0.99893000	
H	3.89919100	-4.70244500	-0.81706300	
H	4.41090700	0.21612500	-0.13800300	
H	1.36508900	0.28771100	-2.71922000	
H	1.39759700	2.32136200	2.10388500	
H	3.26262200	2.39159300	-0.31579900	
H	1.65283400	3.93681800	-1.47657700	
H	-0.32677900	3.66735900	0.84680800	
H	2.51159400	4.76012300	0.63286600	
H	3.30850400	3.44286400	2.95259900	
H	1.06411900	5.74724600	2.20975300	
H	-0.83067600	5.52154200	-0.23286800	
H	0.03925200	2.70946200	-2.83045600	
H	-0.65183200	4.25824600	-2.27903700	
H	5.67253900	-1.89147400	1.29439700	
H	4.68294800	-3.27286600	1.82925000	
H	4.51181400	-1.96153400	-2.01691400	

H 6.59159600 -3.42731000 -0.24041100<br>H -4.25563800 2.91606800 0.21356400<br>H -2.67009100 0.58087500 -1.01769600<br>H -5.94628200 1.45847400 0.74640200<br>H -7.49430400 -0.31861400 1.55189900<br>H -3.21115000 -1.57149300 -0.63396800<br>H -6.66929100 -3.73668300 1.18273000<br>H -3.59986000 -5.14968400 -0.27817100<br>H -3.46970600 -3.72860300 -1.35785900<br>H -2.73381400 -3.67860900 0.26526700<br>H -0.62033000 0.40743300 -1.73153700<br>O 1.72566000 -3.19391000 2.48232500<br>H 1.53293200 -4.12654400 2.27912800<br>O 0.65451200 -2.51224800 1.93882500<br>H 1.06428700 -2.22096800 0.81157100	
**Name**	**6-FMT-O24-H-OOH-P**
Cartesian Coordinates	Energy
C 3.16544700 -2.24018100 0.59218700<br>C 1.70564400 -2.49399100 0.11900700<br>C 1.75794700 -3.92441400 -0.38384700<br>C 2.99364800 -4.42348800 -0.40017500<br>C 4.02335300 -3.44048300 0.09765800<br>C 3.67878900 -0.89414500 0.11901400<br>O 3.47503800 -0.71863600 -1.29156400<br>C 2.17744500 -0.82375100 -1.66121300<br>C 1.28153700 -1.55790200 -0.98963200<br>O 2.96097800 0.07040700 0.83945400<br>C 2.99510600 2.25881100 1.71011100<br>C 3.11341000 1.39853700 0.45813400<br>O 2.06546800 1.69326800 -0.45836000<br>C 2.00336400 3.05276700 -0.86407700<br>C 1.73772700 3.93613800 0.35932900<br>C 2.88206400 3.72683500 1.33469300<br>O 4.13976500 2.02011600 2.49287700<br>O 2.69757700 4.44411800 2.53449300<br>O 1.70810600 5.30597000 0.03545600<br>C 0.94939700 3.14972900 -1.95139200<br>O -0.32153500 2.66646000 -1.50188600<br>C 4.90077800 -3.99969900 1.21742500<br>O 4.96896500 -3.09614500 -0.91713400<br>O 5.69057700 -5.07054700 0.76261700<br>C -1.19024600 3.57299900 -0.99163500<br>C -2.48553800 3.00651500 -0.59496900<br>C -2.78393400 1.70627400 -0.77209200<br>C -4.00528300 1.01021700 -0.39013200<br>O -0.90372300 4.74978700 -0.86762400<br>C -5.14349500 1.66528400 0.12757200<br>C -6.25717700 0.93985000 0.48350500<br>C -6.29891700 -0.46797600 0.32044600<br>C -5.12909400 -1.12661500 -0.18327600<br>C -4.00928500 -0.38634400 -0.54086000<br>O -7.39447900 -1.12852600 0.56608500<br>O -5.21100300 -2.47421100 -0.26080000<br>C -4.15646100 -3.16495000 -0.93085100	Zero-point correction= 0.573682 (Hartree/Particle)<br>Thermal correction to Energy= 0.611460<br>Thermal correction to Enthalpy= 0.612404<br>Thermal correction to Gibbs Free Energy= 0.502610<br>Sum of electronic and zero-point Energies= -2211.470734<br>Sum of electronic and thermal Energies= -2211.432957<br>Sum of electronic and thermal Enthalpies= -2211.432012<br>Sum of electronic and thermal Free Energies= -2211.541806

C -0.14661000 -1.47839400 -1.34824000<br>O -0.95055300 -2.36280600 -1.16034800<br>O -0.55594600 -0.30727200 -1.89684000<br>H 3.18639400 -2.21488000 1.68721900<br>H 0.99779100 -2.39882100 0.94976900<br>H 0.85553600 -4.43389300 -0.70700300<br>H 3.28315200 -5.41598400 -0.73154400<br>H 4.75769700 -0.76903200 0.24527100<br>H 1.95359700 -0.23972500 -2.54911200<br>H 2.08011300 1.95294300 2.24052500<br>H 4.08901400 1.55969700 -0.03108700<br>H 2.96116700 3.36480600 -1.31644000<br>H 0.80054500 3.62035500 0.84157600<br>H 3.81928700 4.04523700 0.84602700<br>H 4.13054700 2.65951000 3.22290700<br>H 2.52833100 5.37050900 2.29849600<br>H 0.81420600 5.51112500 -0.29293600<br>H 1.22293300 2.49954400 -2.78666100<br>H 0.85951400 4.17897100 -2.30637200<br>H 5.52513200 -3.18296900 1.61525200<br>H 4.27096300 -4.38289700 2.02511900<br>H 4.50058200 -2.78715400 -1.70768400<br>H 6.15541700 -4.76046400 -0.03100000<br>H -3.15656900 3.72534800 -0.13657700<br>H -2.03868000 1.07240000 -1.24069000<br>H -5.14460400 2.74493000 0.24078400<br>H -7.15044500 1.41502500 0.87555300<br>H -3.12119600 -0.87717600 -0.93255500<br>H -4.46420500 -4.20928400 -0.95644900<br>H -4.03896300 -2.78304200 -1.94965700<br>H -3.20767800 -3.05897300 -0.39693100<br>H 0.05451100 0.41802800 -1.66425000<br>O -5.57296200 -2.22435500 2.49946900<br>H -5.13786500 -2.69753500 1.76069500<br>O -6.88531900 -2.56562700 2.36075000<br>H -7.23155200 -1.95232700 1.37447900	
**Name**	**10-FMT-C5-H-OOH-P**
Cartesian Coordinates	Energy
C 2.61888400 -0.37426900 0.02942400<br>C 3.96990800 0.13506300 -0.48220600<br>C 5.00122600 -0.52731500 0.34363700<br>C 4.46469600 -1.49550600 1.09913200<br>C 2.95589800 -1.55993100 0.98918400<br>C 1.84281700 0.71343500 0.74559600<br>O 1.75148100 1.90068300 -0.07660300<br>C 2.87519700 2.32209500 -0.66987100<br>C 3.98951500 1.57198800 -0.82153400<br>O 0.54064500 0.27697400 0.94124600<br>C -1.37261900 -0.06671500 2.32202000<br>C -0.08526700 0.75192000 2.13554700<br>O -0.26378000 2.13530900 2.11351400<br>C -1.43656600 2.74189800 1.54655300<br>C -2.69088700 1.87152600 1.67705200<br>C -2.49608500 0.84594600 2.77461900	Zero-point correction= 0.573945 (Hartree/Particle)<br>Thermal correction to Energy= 0.612712<br>Thermal correction to Enthalpy= 0.613656<br>Thermal correction to Gibbs Free Energy= 0.502351<br>Sum of electronic and zero-point Energies= -2211.470638<br>Sum of electronic and thermal Energies= -2211.431871<br>Sum of electronic and thermal Enthalpies= -2211.430927<br>Sum of electronic and thermal Free Energies= -2211.542231

O	-1.22984100	-1.08380200	3.29098900	
O	-3.70658200	0.13335700	2.90673300	
O	-3.77824200	2.73722000	1.93161600	
C	-1.18801500	3.19368200	0.11464400	
O	-1.07394900	2.11573700	-0.79457900	
C	2.50037500	-2.87986800	0.35837800	
O	2.43608200	-1.37605300	2.29585200	
O	1.14410000	-2.81519400	-0.12697700	
C	0.14363400	-2.95225000	0.73933000	
C	-1.19622600	-2.82723600	0.15530300	
C	-1.46495500	-1.95590800	-0.83216100	
O	0.32154200	-3.13953900	1.94073200	
C	-2.81171800	-1.65727200	-1.32338500	
C	-3.80175400	-2.64151100	-1.39137500	
C	-5.08393400	-2.32651700	-1.83314700	
C	-5.38965700	-1.01978800	-2.18912800	
C	-4.40076200	-0.01997800	-2.11284900	
C	-3.11590300	-0.33501600	-1.70093000	
O	-6.63508700	-0.70556800	-2.61220600	
H	1.99141700	-0.70353700	-0.80671100	
H	4.08288100	-0.45635400	-1.58716100	
H	6.05444200	-0.27554700	0.28235100	
H	4.99834600	-2.14891900	1.78233100	
H	2.31600900	1.01560700	1.68733600	
H	2.80996900	3.34340400	-1.03273800	
H	-1.66253800	-0.49332500	1.35311900	
H	0.59503200	0.55911100	2.97248000	
H	-1.60274200	3.63144300	2.16478100	
H	-2.86685800	1.31699000	0.74128400	
H	-2.23396200	1.34094100	3.72264900	
H	-0.80961700	-1.87129900	2.89951800	
H	-3.52311100	-0.65090000	3.44898000	
H	-4.54513100	2.17606300	2.13201000	
H	-0.29061800	3.82473600	0.08651500	
H	-2.04579000	3.79446400	-0.20838700	
H	-0.22234600	1.68155900	-0.62126000	
H	3.09455700	-3.07956700	-0.53512600	
H	2.59431100	-3.70668300	1.06964700	
H	1.71315500	-2.00952700	2.45283300	
H	-1.98474800	-3.33245700	0.70643100	
H	-0.64347900	-1.36983800	-1.24500000	
H	-3.56107800	-3.66750100	-1.12812500	
H	-5.86026500	-3.08080900	-1.90940300	
H	-2.35530600	0.43880600	-1.62407500	
H	-6.65997600	0.24857800	-2.79801000	
C	5.14015800	2.21860000	-1.47753800	
O	5.21463700	3.38283700	-1.79776000	
O	6.15297500	1.34688300	-1.70152700	
H	6.85874600	1.84955500	-2.14467400	
O	-4.83968900	1.21803400	-2.47948500	
C	-3.97368600	2.31438700	-2.22506100	
H	-3.04041000	2.22137300	-2.78934300	
H	-4.51632000	3.20416200	-2.54278000	
H	-3.74388500	2.37632700	-1.15534600	
O	4.80813800	-2.35922800	-2.10245900	

H 5.72364700 -2.15099200 -2.36225900<br>O 4.07882500 -1.30198000 -2.58399800	
**Name**	**10-FMT-O24-H-OOH-P**
Cartesian Coordinates	Energy
C 3.06174600 -0.03735000 -0.86602500<br>C 4.25279000 0.89308000 -1.20521900<br>C 5.43998300 -0.04294100 -1.24958700<br>C 5.06874700 -1.32053800 -1.23854300<br>C 3.57636200 -1.50302600 -1.07529000<br>C 2.56645100 0.12282500 0.56071400<br>O 2.34720200 1.50564300 0.89502400<br>C 3.36517700 2.34039100 0.61708000<br>C 4.32867000 2.08357100 -0.28282800<br>O 1.33239500 -0.50845000 0.65770500<br>C -0.16844700 -2.01632700 1.72996400<br>C 1.03319300 -1.07499200 1.93383900<br>O 0.85880000 -0.09550200 2.91253100<br>C -0.40693400 0.54678400 3.12765200<br>C -1.59592800 -0.32929000 2.73037000<br>C -1.20023400 -1.78894100 2.81740500<br>O 0.20271600 -3.37903800 1.74917800<br>O -2.36145700 -2.55594000 2.58194600<br>O -2.66843000 0.00015200 3.58822200<br>C -0.45064800 1.92027800 2.47307900<br>O -0.51679100 1.86117700 1.06046400<br>C 2.93730500 -2.10557100 -2.33136900<br>O 3.38472700 -2.33699400 0.05644100<br>O 1.51124200 -1.88661800 -2.38449800<br>C 0.70979200 -2.68023900 -1.67939100<br>C -0.71777700 -2.33029000 -1.76595800<br>C -1.13450200 -1.05394500 -1.72955200<br>O 1.11938600 -3.61157700 -0.99579700<br>C -2.53868100 -0.65495900 -1.61160000<br>C -3.56618100 -1.40963000 -2.21540600<br>C -4.88203600 -1.02914100 -2.07291300<br>C -5.23585800 0.08957700 -1.27902600<br>C -4.18131600 0.85116200 -0.67693400<br>C -2.85198300 0.48689100 -0.86016800<br>O -6.48790100 0.37418800 -1.05950900<br>H 2.20466200 0.18180500 -1.51038100<br>H 4.11029300 1.29523000 -2.21992300<br>H 6.45668600 0.32420500 -1.34125000<br>H 5.72554900 -2.18376400 -1.28596400<br>H 3.28112300 -0.27054700 1.29328000<br>H 3.32848200 3.26570200 1.18397200<br>H -0.64691000 -1.76542600 0.77587600<br>H 1.90119700 -1.65825700 2.25998800<br>H -0.46262900 0.68366900 4.21415000<br>H -1.88192200 -0.12794100 1.68540700<br>H -0.77890000 -2.01829400 3.80833400<br>H 0.43811700 -3.66306900 0.84841900<br>H -2.08604200 -3.48161200 2.48573600<br>H -3.35336900 -0.67707300 3.46007600<br>H 0.42188100 2.50091500 2.79807500	Zero-point correction= 0.575012 (Hartree/Particle)<br>Thermal correction to Energy= 0.613130<br>Thermal correction to Enthalpy= 0.614074<br>Thermal correction to Gibbs Free Energy= 0.503743<br>Sum of electronic and zero-point Energies= -2211.471946<br>Sum of electronic and thermal Energies= -2211.433829<br>Sum of electronic and thermal Enthalpies= -2211.432885<br>Sum of electronic and thermal Free Energies= -2211.543215

H -1.35579800 2.43429200 2.81619100<br>H 0.36594900 1.60486300 0.74507200<br>H 3.31227500 -1.59420600 -3.21959300<br>H 3.14512700 -3.17776000 -2.40283600<br>H 2.65746500 -2.96096300 -0.10886300<br>H -1.40402200 -3.16561300 -1.65556800<br>H -0.38844800 -0.26034100 -1.69117600<br>H -3.31043000 -2.27233800 -2.82377200<br>H -5.69262800 -1.58009000 -2.53852200<br>H -2.05331700 1.05610100 -0.38846700<br>C 5.35160800 3.13172200 -0.45628000<br>O 5.48370800 4.12100000 0.22823400<br>O 6.16273200 2.87603700 -1.51017100<br>H 6.79467800 3.61408700 -1.56501100<br>O -4.58108900 1.92776700 0.04091300<br>C -3.66693500 2.46379000 0.99905100<br>H -2.78421100 2.89421500 0.51647700<br>H -4.22315200 3.23637300 1.52879900<br>H -3.36076700 1.67991200 1.70068100<br>O -5.57214700 2.74670300 -2.41413900<br>H -5.10547300 2.97852800 -1.58431600<br>O -6.84839600 2.50185300 -1.99977400<br>H -6.75522000 1.46683900 -1.39743700	
**Name**	**6-FMT-ANION**
Cartesian Coordinates	Energy
C 1.97722000 -2.40378000 0.67975600<br>C 0.43975400 -2.50330400 0.78709600<br>C 0.23619800 -4.00185100 0.92701600<br>C 1.33212400 -4.69987000 0.62292100<br>C 2.47263900 -3.79921300 0.21462200<br>C 2.48306200 -1.25694100 -0.17451900<br>O 1.79628300 -1.11219700 -1.41104000<br>C 0.45448400 -1.35880900 -1.42132300<br>C -0.23914300 -1.97255000 -0.45446700<br>O 2.36503200 -0.05538600 0.56240900<br>C 3.40344500 1.88558200 1.48907100<br>C 3.45999100 0.82098700 0.38694700<br>O 3.52653900 1.32748600 -0.92175100<br>C 2.79788500 2.49944900 -1.31928500<br>C 2.54779800 3.46499800 -0.15152800<br>C 3.62290500 3.26936200 0.90357400<br>O 4.38822800 1.54039900 2.44953800<br>O 3.48159500 4.20203700 1.96034500<br>O 2.57646600 4.80834300 -0.59967100<br>C 1.56110000 2.15631900 -2.14943300<br>O 0.36412300 1.93942200 -1.37602100<br>C 3.81267200 -4.14650300 0.85267000<br>O 2.71534200 -3.88582900 -1.19677200<br>O 4.28778100 -5.41390400 0.42326100<br>C -0.39695200 3.01790500 -1.13563900<br>C -1.68109300 2.75148300 -0.48207600<br>C -2.26898700 1.54357600 -0.42640700<br>C -3.58743000 1.29485500 0.16561100<br>O -0.04650700 4.15417500 -1.43865200	Zero-point correction= 0.549925 (Hartree/Particle)<br>Thermal correction to Energy= 0.587225<br>Thermal correction to Enthalpy= 0.588169<br>Thermal correction to Gibbs Free Energy= 0.479781<br>Sum of electronic and zero-point Energies= -2060.224295<br>Sum of electronic and thermal Energies= -2060.186995<br>Sum of electronic and thermal Enthalpies= -2060.186051<br>Sum of electronic and thermal Free Energies= -2060.294440

C -4.16295200 2.16035600 1.10418200<br>C -5.43470300 1.90636700 1.61036400<br>C -6.14449000 0.79355200 1.17713700<br>C -5.57480500 -0.08783900 0.24154600<br>C -4.30157500 0.15643200 -0.25220300<br>O -7.39485300 0.55670700 1.67324000<br>O -6.37089100 -1.14331600 -0.10212900<br>C -5.84795800 -2.06801400 -1.05368900<br>C -1.72594400 -2.15311000 -0.61830500<br>O -2.37264500 -2.46959600 0.42127500<br>O -2.23911300 -1.98461800 -1.76334500<br>H 2.38522800 -2.24115600 1.68306000<br>H 0.06546800 -1.97167800 1.66967300<br>H -0.71961200 -4.42788600 1.21530600<br>H 1.41962000 -5.78274500 0.60839900<br>H 3.52772800 -1.41085800 -0.45965500<br>H -0.00193400 -1.00662800 -2.34111700<br>H 2.40901100 1.86891400 1.95396100<br>H 4.38893200 0.25200200 0.50050300<br>H 3.48593600 3.00772700 -2.00325100<br>H 1.58600900 3.26365300 0.33805800<br>H 4.62493800 3.34650600 0.45736900<br>H 4.26367600 2.11117200 3.22579700<br>H 3.52447400 5.09447700 1.57709400<br>H 1.71977400 4.97784500 -1.03393100<br>H 1.73293200 1.22092400 -2.68337400<br>H 1.37216100 2.95535500 -2.86828900<br>H 4.54221400 -3.36036900 0.61009100<br>H 3.70059100 -4.19898900 1.93793200<br>H 1.86423500 -3.85451700 -1.66638800<br>H 4.31835800 -5.39695900 -0.54824000<br>H -2.17060300 3.64694800 -0.11109700<br>H -1.77329400 0.68636500 -0.88469100<br>H -3.61539500 3.02717200 1.46133400<br>H -5.88838900 2.56397400 2.34583700<br>H -3.84369100 -0.52255900 -0.96630400<br>H -7.74922400 -0.25402200 1.26476000<br>H -6.62781600 -2.81417600 -1.20240600<br>H -5.63003800 -1.56265900 -2.00035700<br>H -4.94180700 -2.54426400 -0.66738600	
**Name**	**6-FMT-DIANION**
Cartesian Coordinates	Energy
C 0.05754400 -2.45873000 0.50043200<br>C -0.80230900 -1.63758100 -0.49313900<br>C -1.96235100 -2.57618400 -0.76576500<br>C -1.76369200 -3.80244800 -0.27929100<br>C -0.42217600 -3.93430000 0.39855600<br>C 1.54548800 -2.28305000 0.28300100<br>O 1.92436800 -2.44684800 -1.07834700<br>C 1.18594400 -1.72937500 -1.97959800<br>C -0.05995500 -1.29007800 -1.76503400<br>O 1.88950400 -0.98619200 0.72830600<br>C 3.65811400 0.19273100 1.82438800<br>C 3.26883500 -0.68465400 0.62477400	Zero-point correction= 0.536409 (Hartree/Particle)<br>Thermal correction to Energy= 0.573325<br>Thermal correction to Enthalpy= 0.574270<br>Thermal correction to Gibbs Free Energy= 0.465875<br>Sum of electronic and zero-point Energies= -2059.778150<br>Sum of electronic and thermal Energies= -2059.741234<br>Sum of electronic and thermal Enthalpies= -2059.740290<br>Sum of electronic and thermal Free Energies= -2059.848685

O	3.58665900	-0.14757600	-0.63574200	
C	3.57646700	1.25997900	-0.91514800	
C	3.65015600	2.12328900	0.34703000	
C	4.47754100	1.39468900	1.39147000	
O	4.35628600	-0.64338500	2.73377200	
O	4.68698700	2.19716900	2.54036100	
O	4.25445400	3.37077000	0.05645500	
C	2.44715400	1.63151600	-1.86966600	
O	1.18304700	1.83944200	-1.22075300	
C	-0.47608200	-4.58153300	1.77754700	
O	0.44975600	-4.78613900	-0.35736000	
O	-0.92287700	-5.92806000	1.71416400	
C	0.88389500	3.10101400	-0.84390400	
C	-0.44726100	3.28591600	-0.29807800	
C	-1.37662500	2.29828200	-0.25634300	
C	-2.72106000	2.38003000	0.26095000	
O	1.69787500	4.02098100	-0.95478700	
C	-3.26216400	3.54164400	0.84094400	
C	-4.55655000	3.55565600	1.33054900	
C	-5.40714200	2.41353200	1.28071100	
C	-4.83502400	1.23726200	0.67443000	
C	-3.54520500	1.22687000	0.18894200	
O	-6.60947300	2.41312100	1.74442500	
O	-5.68359100	0.15401100	0.63201500	
C	-5.16089600	-1.04132900	0.07322400	
C	-0.74150800	-0.46485900	-2.82707600	
O	-1.94359500	-0.14078100	-2.60993300	
O	-0.08386100	-0.14276200	-3.86009500	
H	-0.15682400	-2.11376200	1.51776100	
H	-1.15914700	-0.71594400	-0.01902800	
H	-2.83575900	-2.25636800	-1.32580100	
H	-2.43879300	-4.64771100	-0.38156600	
H	2.13900700	-3.02806900	0.82171800	
H	1.72852300	-1.56215000	-2.90408000	
H	2.74166800	0.56856200	2.29708000	
H	3.83945300	-1.61799600	0.67827000	
H	4.50817700	1.43264300	-1.46686000	
H	2.65255100	2.28949600	0.77722500	
H	5.44115900	1.07636300	0.96715700	
H	4.45568900	-0.16196700	3.57172200	
H	5.12870300	3.01474400	2.25489200	
H	3.56943600	3.91953400	-0.37143900	
H	2.28282100	0.81143800	-2.57221100	
H	2.71874000	2.53188100	-2.42438500	
H	0.52123500	-4.52774400	2.23736000	
H	-1.18462600	-4.04166400	2.40982100	
H	0.44869400	-4.49604900	-1.28548000	
H	-0.32935500	-6.40401000	1.10941000	
H	-0.64213800	4.29105200	0.06235100	
H	-1.09328400	1.32914100	-0.66472500	
H	-2.66061800	4.44445000	0.91249800	
H	-4.96409500	4.45811100	1.78015800	
H	-3.13481000	0.33049100	-0.26733200	
H	-5.96240200	-1.77875300	0.12797900	
H	-4.86936800	-0.89135700	-0.97325300	

H -4.29581000 -1.39408700 0.64748000	
**Name**	**10-FMT-ANION**
Cartesian Coordinates	Energy
C -2.64612900 0.61269300 0.56378700	Zero-point correction= 0.549381 (Hartree/Particle)
C -3.83301300 1.50135000 0.11158000	Thermal correction to Energy= 0.586344
C -4.97134400 1.06336900 1.00306400	Thermal correction to Enthalpy= 0.587288
C -4.55668500 0.28818700 2.00294900	Thermal correction to Gibbs Free Energy= 0.478773
C -3.08195300 -0.03077500 1.92459900	Sum of electronic and zero-point Energies= -2060.232817
C -2.30936400 -0.46355300 -0.45344700	Sum of electronic and thermal Energies= -2060.195853
O -2.17700700 0.07097300 -1.77207400	Sum of electronic and thermal Enthalpies= -2060.194909
C -3.22984500 0.82746600 -2.19180900	Sum of electronic and thermal Free Energies= -2060.303424
C -4.07860000 1.46404700 -1.37444600	
O -1.05989000 -0.99167800 -0.10580300	
C 0.43782800 -2.79552200 0.26184000	
C -0.78199100 -2.31700600 -0.53530500	
O -0.60036700 -2.40566800 -1.92617500	
C 0.69354100 -2.11910600 -2.48743600	
C 1.83131100 -2.85844100 -1.75753500	
C 1.28945100 -3.69920900 -0.61429100	
O 0.04688900 -3.51283400 1.41765600	
O 2.39863200 -4.20820500 0.10758100	
O 2.51571200 -3.65139600 -2.71963200	
C 0.95065600 -0.62598900 -2.64438500	
O 1.19108900 0.06393200 -1.42554700	
C -2.31791200 0.60358600 3.09429500	
O -2.97992000 -1.45598600 1.95554500	
O -0.90210800 0.73642700 2.83135100	
C -0.11759300 -0.33582000 2.89671200	
C 1.26087700 -0.12886900 2.45602500	
C 1.64320400 0.95599500 1.75686700	
O -0.53038500 -1.43528000 3.27543100	
C 2.95639700 1.16286400 1.14935900	
C 3.89941700 0.13555400 1.01768300	
C 5.12559800 0.38076900 0.40682100	
C 5.41799100 1.64892500 -0.08062500	
C 4.47422300 2.68633400 0.03247000	
C 3.25079600 2.44082500 0.63694600	
O 6.62213000 1.87923400 -0.67867400	
H -1.73965100 1.21508000 0.67813500	
H -3.60052900 2.54606900 0.36475600	
H -5.99015700 1.40837100 0.85488800	
H -5.16806300 -0.11836800 2.80386500	
H -3.07029900 -1.25244200 -0.49622000	
H -3.28411600 0.88122000 -3.27411600	
H 1.04411100 -1.92351900 0.54715500	
H -1.64968800 -2.94804200 -0.31702800	
H 0.62896400 -2.53714300 -3.49659600	
H 2.53234500 -2.13017800 -1.32471500	
H 0.68321400 -4.52745000 -1.00865500	
H -0.07828100 -2.87956400 2.15117800	
H 2.05361500 -4.69965800 0.87261400	
H 3.32101800 -3.99960700 -2.30229600	
H 0.11244200 -0.17459100 -3.18795600	
H 1.85614500 -0.50900700 -3.24829700	

H	0.38577300	0.00895200	-0.87540600	
H	-2.64865900	1.63383200	3.23055400	
H	-2.47142800	0.03801000	4.01746400	
H	-2.12486100	-1.71059200	2.34786300	
H	1.92040400	-0.96920200	2.65283600	
H	0.92096700	1.75686600	1.60188500	
H	3.67858700	-0.86686400	1.37302200	
H	5.86397100	-0.40720600	0.29245900	
H	2.51044200	3.23017800	0.72268100	
H	6.66350400	2.80988600	-0.96613000	
C	-5.16635800	2.31809000	-1.97177400	
O	-5.45713500	2.15808800	-3.19317300	
O	-5.71672200	3.15890800	-1.20359300	
O	4.87716400	3.87880300	-0.49481900	
C	3.95906900	4.96691200	-0.42332800	
H	3.72619700	5.20462300	0.61940300	
H	4.46172300	5.81162400	-0.89270500	
H	3.04025300	4.73012700	-0.96893000	
**Name**				**10-FMT-DIANION**
Cartesian Coordinates				Energy
C	2.59840900	-0.61333700	0.55749400	Zero-point correction= 0.536929 (Hartree/Particle)
C	3.78278200	-1.50084000	0.09594100	Thermal correction to Energy= 0.573145
C	4.92252300	-1.07550600	0.99201700	Thermal correction to Enthalpy= 0.574089
C	4.50952200	-0.31024000	2.00020900	Thermal correction to Gibbs Free Energy= 0.467920
C	3.03486300	0.01014000	1.92766400	Sum of electronic and zero-point Energies= -2059.782037
C	2.26933200	0.47761900	-0.44681700	Sum of electronic and thermal Energies= -2059.745821
O	2.13797700	-0.03822000	-1.77343900	Sum of electronic and thermal Enthalpies= -2059.744877
C	3.18637200	-0.79642700	-2.20033200	Sum of electronic and thermal Free Energies= -2059.851046
C	4.02994300	-1.44802000	-1.38935500	
O	1.02227000	1.00900300	-0.09751300	
C	-0.46193600	2.81800200	0.29439700	
C	0.75726000	2.34330100	-0.50503000	
O	0.58102100	2.45640800	-1.89510400	
C	-0.71706800	2.19890800	-2.46074300	
C	-1.84006200	2.96144000	-1.73111400	
C	-1.28692900	3.76222500	-0.56422100	
O	-0.07177100	3.49134000	1.47626200	
O	-2.38814700	4.27968100	0.16401900	
O	-2.48724400	3.79403300	-2.68570800	
C	-1.00663600	0.71213700	-2.62410900	
O	-1.27248900	0.02257200	-1.41028900	
C	2.27075900	-0.64126900	3.08945300	
O	2.93098200	1.43452800	1.97963000	
O	0.85911600	-0.78066900	2.82750000	
C	0.06626100	0.29694500	2.88174800	
C	-1.28985300	0.09897800	2.42590700	
C	-1.70184500	-1.03277700	1.79359400	
O	0.49288100	1.39336400	3.27837800	
C	-2.98016200	-1.25783800	1.17269900	
C	-3.96007900	-0.25484500	1.04479400	
C	-5.15332400	-0.50596400	0.39346600	
C	-5.45990500	-1.77599400	-0.17757500	
C	-4.44063000	-2.78875000	-0.03657400	
C	-3.24939200	-2.53173200	0.60573000	

O	-6.56548600	-2.02080000	-0.78724100	
H	1.68890900	-1.21279600	0.66298200	
H	3.54696800	-2.54751700	0.33774900	
H	5.94050300	-1.42122900	0.83971900	
H	5.12224900	0.08609900	2.80530800	
H	3.03524100	1.26241100	-0.47744800	
H	3.24207500	-0.83771600	-3.28312400	
H	-1.08568400	1.94839500	0.54628700	
H	1.62916600	2.96367800	-0.27290400	
H	-0.64067600	2.61821000	-3.46853300	
H	-2.56885000	2.24758900	-1.32161300	
H	-0.65950500	4.58563600	-0.93514100	
H	0.04390600	2.82999200	2.18851900	
H	-2.03647300	4.72036600	0.95653500	
H	-3.29489000	4.14681600	-2.27701500	
H	-0.17458800	0.24381500	-3.16290200	
H	-1.90975900	0.61794000	-3.23542900	
H	-0.46564600	0.04353600	-0.86005400	
H	2.61020700	-1.67040800	3.21495300	
H	2.42629300	-0.08515300	4.01829200	
H	2.07529100	1.67611900	2.38183800	
H	-1.92942800	0.96873900	2.54321000	
H	-0.99272200	-1.85781500	1.72074900	
H	-3.77732400	0.73864800	1.44791100	
H	-5.90081700	0.27725300	0.29181400	
H	-2.48945400	-3.30358700	0.69199700	
C	5.11432500	-2.30037200	-1.99498600	
O	5.40547600	-2.13034800	-3.21505400	
O	5.66236500	-3.14991100	-1.23477400	
O	-4.77468600	-3.99869900	-0.59936300	
C	-3.81759200	-5.04197600	-0.49856000	
H	-3.60935300	-5.28353000	0.54992100	
H	-4.26090500	-5.90742700	-0.99169400	
H	-2.88449700	-4.76918600	-1.00453000	

References

1. M. G. Evans and M. Polanyi, *Trans. Faraday Soc.*, 1935, **31**, 875-894.
2. H. Eyring, *J. Chem. Phys.*, 1935, **3**, 107-115.
3. D. G. Truhlar, W. L. Hase and J. T. Hynes, *J. Phys. Chem.*, 1983, **87**, 2664-2682.
4. T. Furuncuoglu, I. Ugur, I. Degirmenci and V. Aviyente, *Macromolecules*, 2010, **43**, 1823-1835.
5. E. Vélez, J. Quijano, R. Notario, E. Pabón, J. Murillo, J. Leal, E. Zapata and G. Alarcón, *J. Phys. Org. Chem.*, 2009, **22**, 971-977.
6. E. Pollak and P. Pechukas, *J. Am. Chem. Soc.*, 1978, **100**, 2984-2991.
7. A. Fernández-Ramos, B. A. Ellingson, R. Meana-Pañeda, J. M. Marques and D. G. Truhlar, *Theor. Chem. Acc.*, 2007, **118**, 813-826.
8. C. Eckart, *Phy. Rev.*, 1930, **35**, 1303.
9. R. A. Marcus, *Annu. Rev. Phys. Chem.*, 1964, **15**, 155-196.
10. R. A. Marcus, *Rev. Mod. Phys.*, 1993, **65**, 599.
11. Y. Lu, A. Wang, P. Shi and H. Zhang, *PloS one*, 2017, **12**, e0169773.
12. Y. Lu, A. Wang, P. Shi, H. Zhang and Z. Li, *PloS one*, 2015, **10**, e0133259.
13. S. F. Nelsen, S. C. Blackstock and Y. Kim, *J. Am. Chem. Soc.*, 1987, **109**, 677-682.
14. S. F. Nelsen, M. N. Weaver, Y. Luo, J. R. Pladziewicz, L. K. Ausman, T. L. Jentzsch and J. J. O'Konek, *J. Phys. Chem. A*, 2006, **110**, 11665-11676.
15. A. Galano and J. R. Alvarez-Idaboy, *J. Comput. Chem.*, 2013, **34**, 2430-2445.
16. F. C. Collins and G. E. Kimball, *J. Colloid Sci.* , 1949, **4**, 425-437.
17. M. Von Smoluchowski, *Z. Phys. Chem*, 1917, **92**, 129-168.
18. D. G. Truhlar, *J. Chem. Educ.*, 1985, **62**, 104.
19. A. Einstein, *Ann. Phys.*, 1905, **17**, 549-560.
20. G. G. Stokes, *Mathematical and Physical Papers*, University Press, Cambridge, 1905.
21. A. Galano and J. Raúl Alvarez-Idaboy, *Int. J. Quantum Chem.*, 2019, **119**, e25665.
22. Q. V. Vo, T. V. Gon, M. V. Bay and A. Mechler, *J. Phys. Chem. B*, 2019, **123**, 10672-10679.
23. Q. V. Vo and A. Mechler, *J. Chem. Inf. Model.*, 2020, **60**, 316-321.
24. Y. Okuno, *Chem.: Eur. J.*, 1997, **3**, 212-218.
25. S. Benson, *The foundations of chemical kinetics:* , Malabar, Florida, 1982.
26. C. Iuga, J. R. Alvarez-Idaboy and A. Vivier-Bunge, *J. Phys. Chem. B*, 2011, **115**, 12234-12246.
27. J. R. Alvarez-Idaboy, L. Reyes and N. Mora-Diez, *Org. Biomol. Chem.*, 2007, **5**, 3682-3689.
28. T. H. Le, T. T. Tran and L. K. Huynh, *Chemom. Intell. Lab. Syst.*, 2018, **172**, 10-16.
29. E. Dzib, J. L. Cabellos, F. Ortíz-Chi, S. Pan, A. Galano and G. Merino, *Int. J. Quantum Chem.*, 2019, **119**, e25686.

30. E. Dzib, J. L. Cabellos, F. Ortiz-Chi, S. Pan, A. Galano and G. Merino, *Eyringpy 1.0.2*, 2018, Cinvestav, Mérida, Yucatán.
